# Supplementary material for: Fungal community assembly in drought-stressed sorghum shows stochasticity, selection, and universal ecological dynamics
Source: Nat Commun. 2020 Jan 7;11:34. doi: 10.1038/s41467-019-13913-9 (PMC6946711; doi:10.1038/s41467-019-13913-9)
Supplement: Supplementary file 1 — Supplementary Information [file 41467_2019_13913_MOESM1_ESM.pdf]

Supplementary Information for “Fungal community assembly in drought stressed sorghum shows stochasticity, selection, and universal ecological dynamics” by Gao et al.

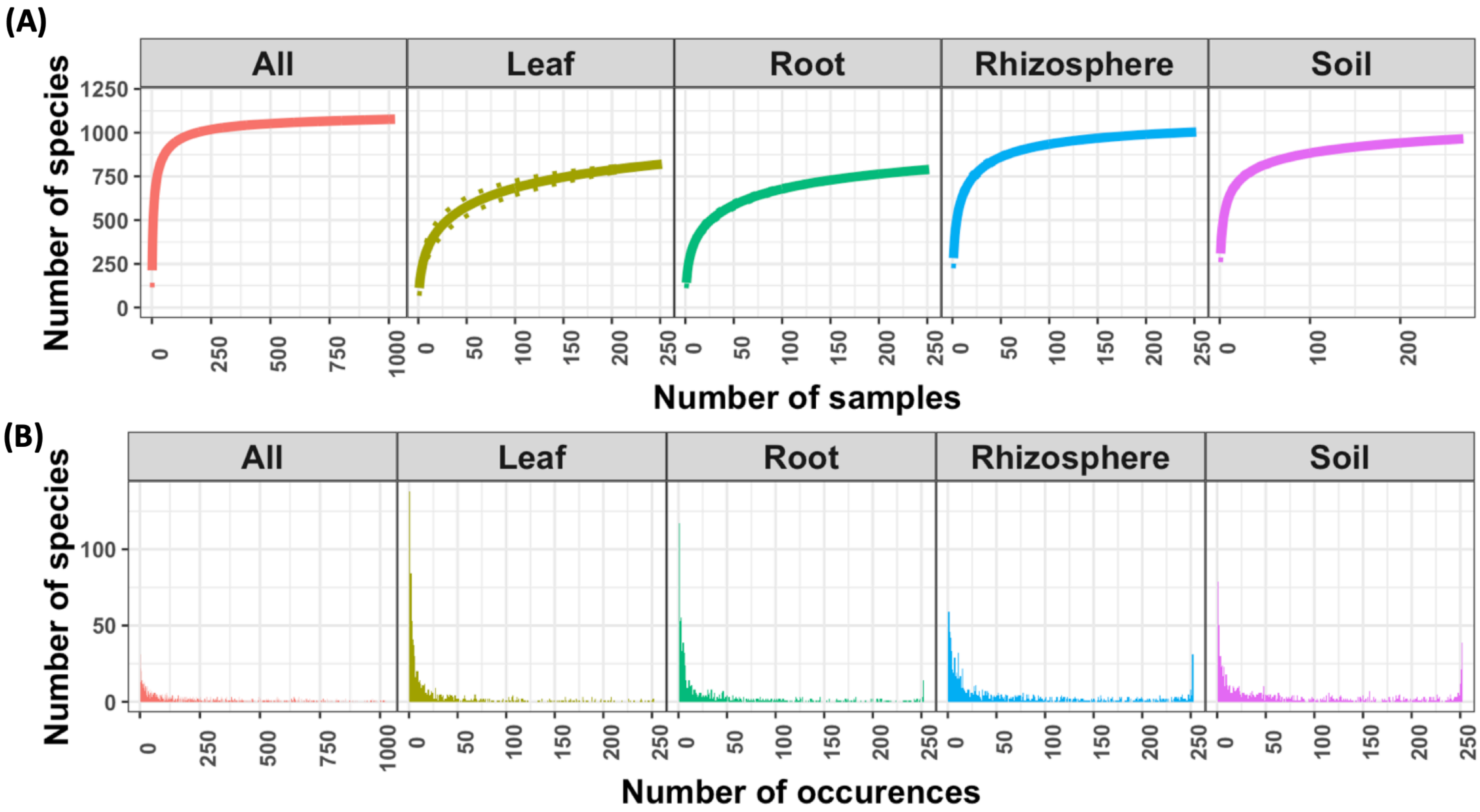

Supplementary Fig. 1 Operational taxonomic unit (OTU) sampling and occurrence. (A) The fungal OTU accumulation curve for all 1026 samples and for 252 leaf, 252 root, 252 rhizosphere and 270 soil samples. For all 1026 samples, the OTU accumulation curve reached a plateau of 986 of 1070 taxa after just 250 samples. (B) The frequency of fungal OTUs found in as few as 1 to as many as 1026 of all 1026 communities sampled. For specific compartments, many species were found in all 252 or 270 samples. The the y axis is the same for all and separate compartments. Source data are provided as a Source Data file

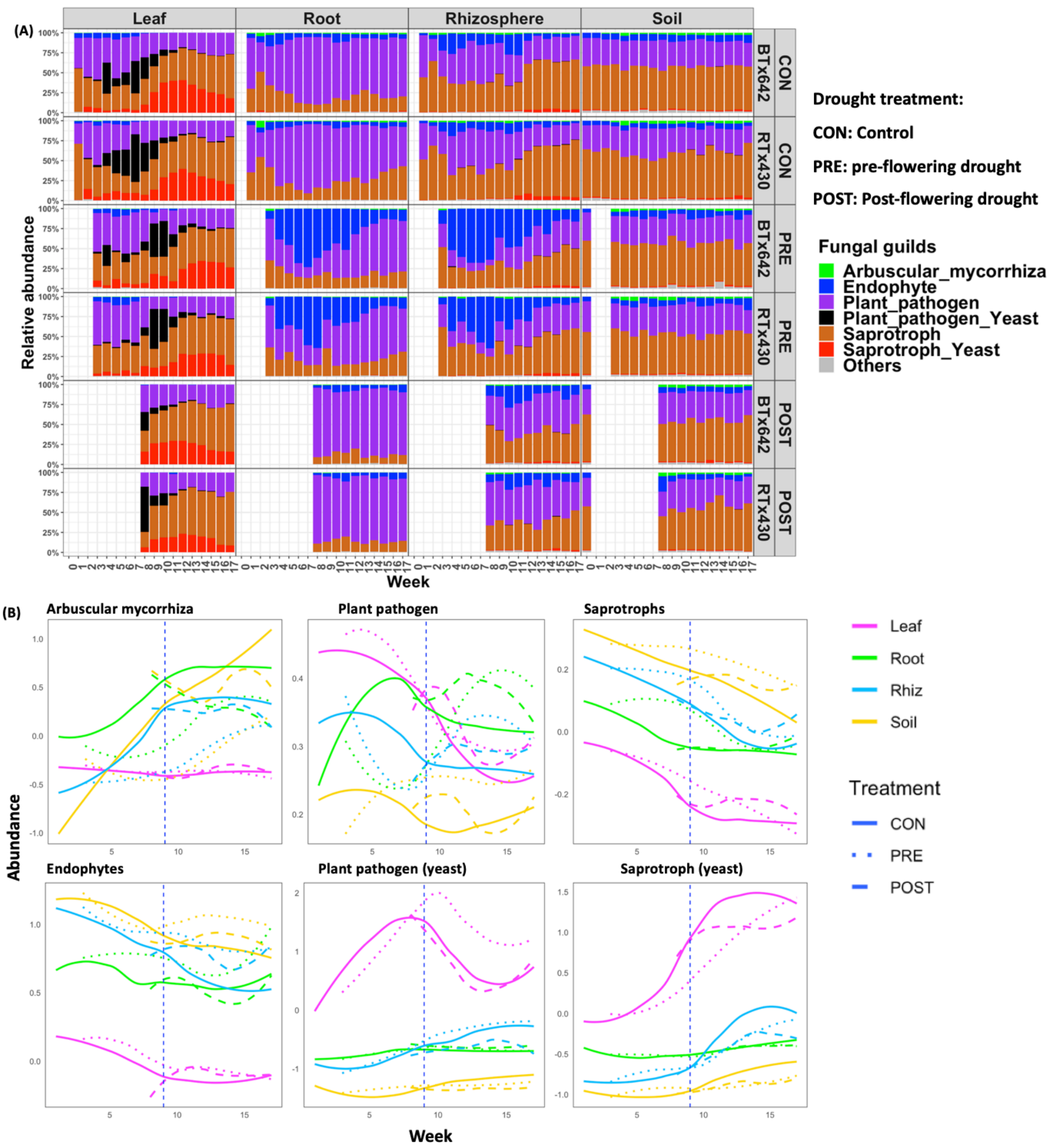

Supplementary Fig. 2 Fungal guild abundance. (A) The temporal compositional variance of fungal functional guilds in the four compartments, three treatments and two sorghum cultivars (RTx430, BTx642). (B) Regression curve of every fungal guild for each treatment in each compartment. Source data are provided as a Source Data file.

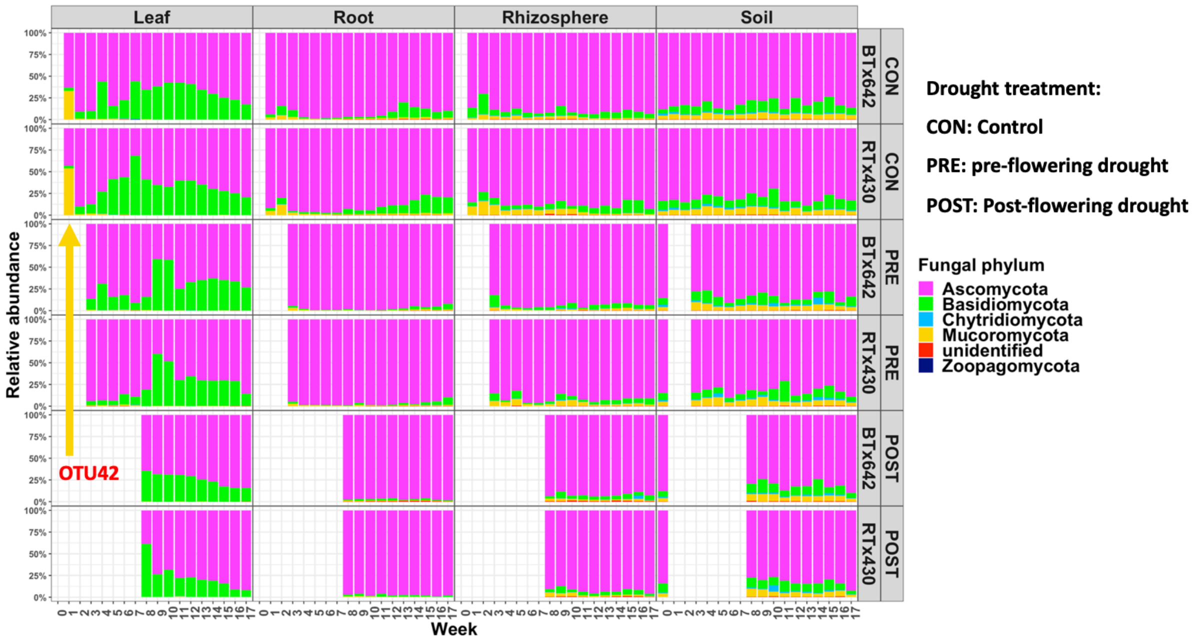

Supplementary Fig. 3 Fungal phyla abundance. The temporal compositional variance of fungal phyla in the four compartments, three treatments and two sorghum cultivars (RTx430, BTx642). Note the rapid boom and vanish of OTU42 (*Actinomucor*, Mucoromycota) in first week leaves. Source data are provided as a Source Data file.

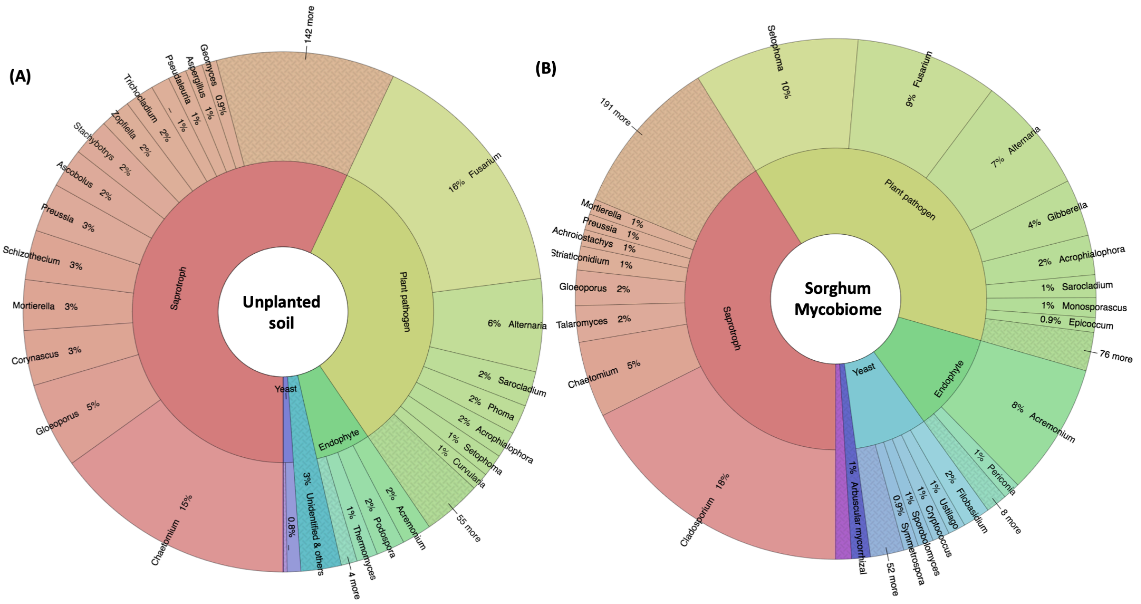

Supplementary Fig. 4 Krona graphs showing fungal guild and genus abundance. Functional affiliation and relative abundance of mycobiome of (A) unplanted soil and (B) sorghum mycobiome. The interactive version of these two Krona figures can be found at online Supplementary Software 1. Source data are provided as a Source Data file.

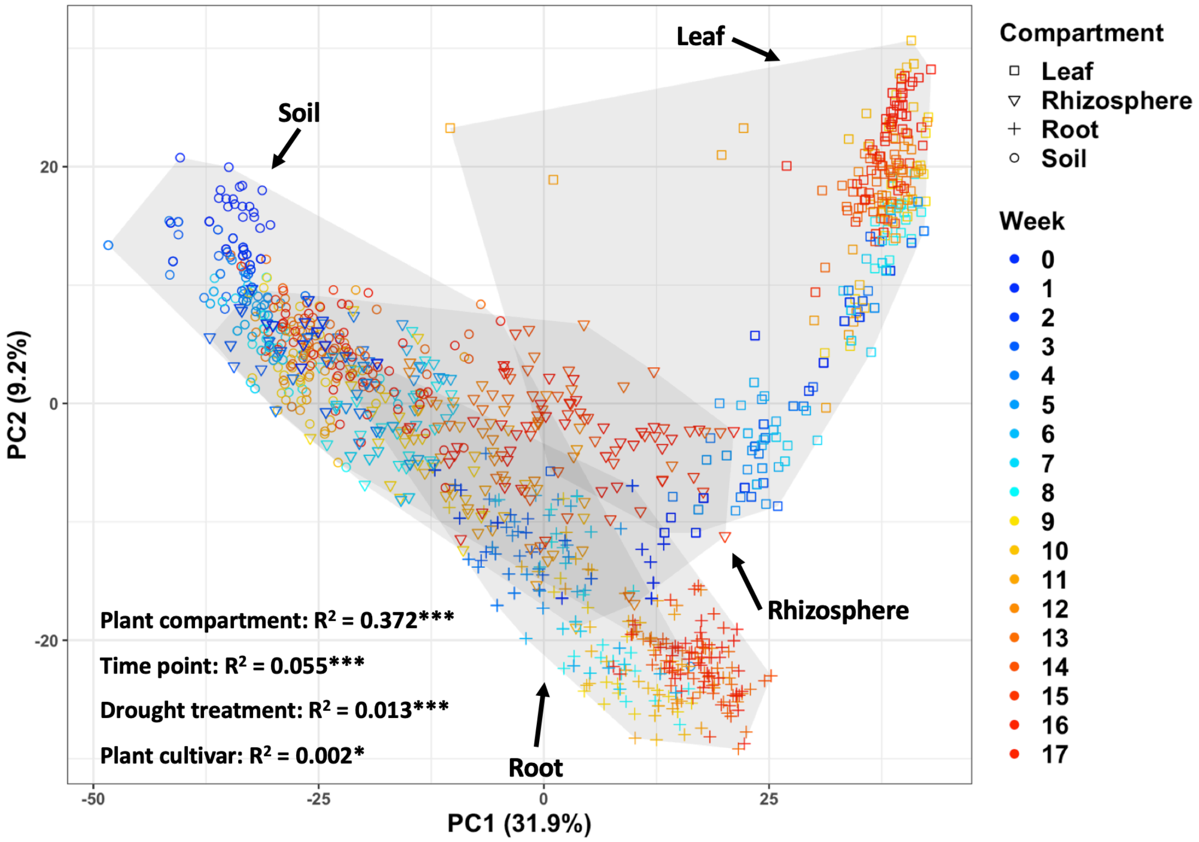

Supplementary Fig. 5 Fungal community dynamics detected from a compositional dataset (dataset 2). Principal component (PC) analysis of fungal community Aitchison distance with permutational analysis of variance (PERM ANOVA) showing significant association of fungal community composition with, in order of importance, compartment, time period, drought treatment and sorghum cultivar (**P* < 0.05; *** *P* < 0.001). Note the result of principal coordinate (PCo) analysis of Bray-Curtis dissimilarity (dataset 1) is presented in Fig. 2A. Source data are provided as a Source Data file.

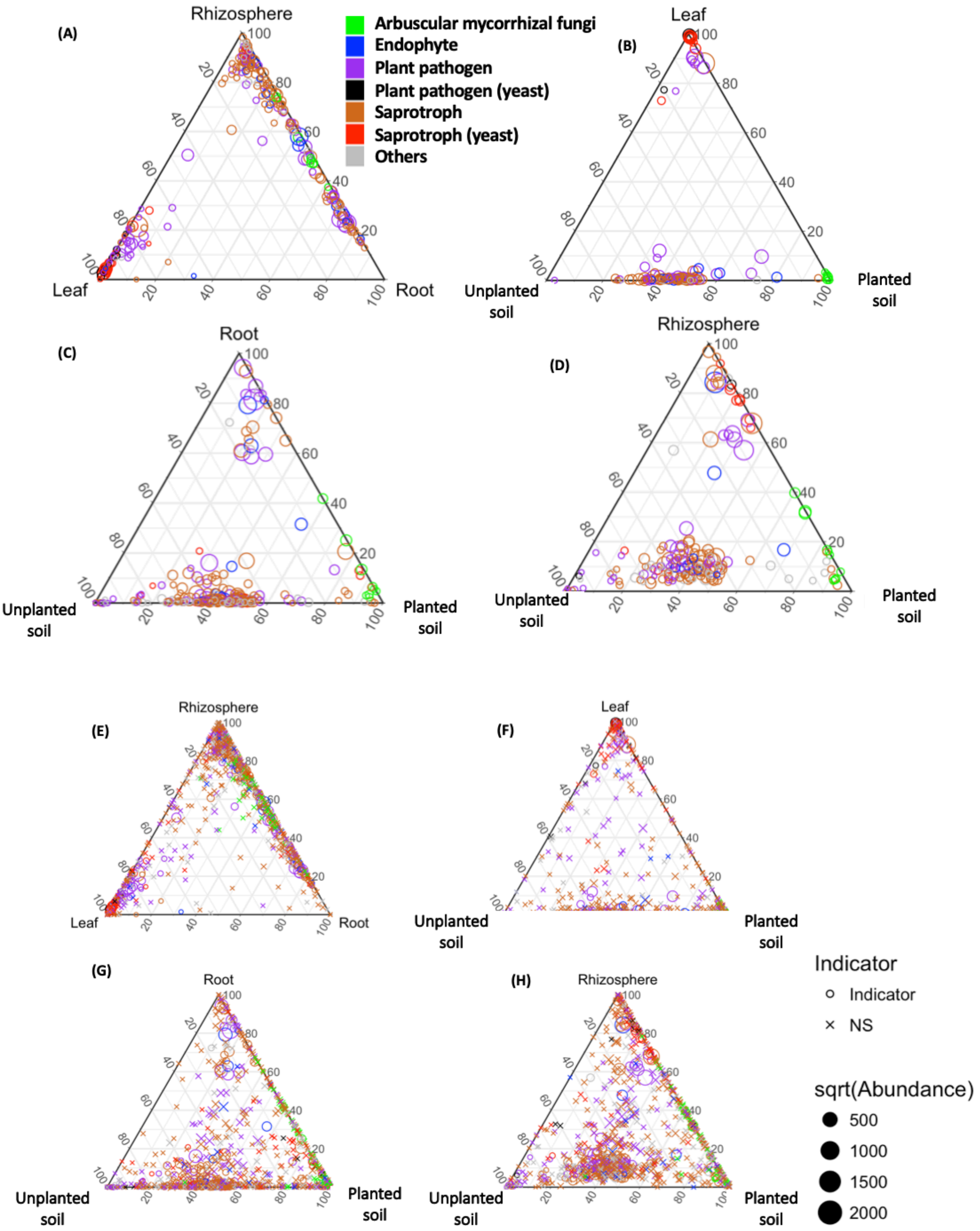

Supplementary Fig. 6 Fungal functional guilds affected by sorghum compartment showing significantly biased occurrences of yeasts and pathogenic fungi toward leaf, and biased occurrences of arbuscular mycorrhizal and saprotrophic fungi toward root, rhizosphere and planted soil. Ternary plot demonstrating the distribution fungal operational taxonomic units (OTUs) (A) among leaf, root and rhizosphere, (B) among unplanted soil, planted soil and leaf, (C) among unplanted soil, planted soil and root, and (D) among unplanted soil, planted soil and rhizosphere. The circle size corresponds to OTU abundance (sqrt transformed). To simplify, the plots A-D only kept OTUs whose abundances were significantly (*P* < 0.0001) different among different compartments. Plots containing both significant and non-significant (NS) OTUs are shown in plots E-H. Source data are provided as a Source Data file.

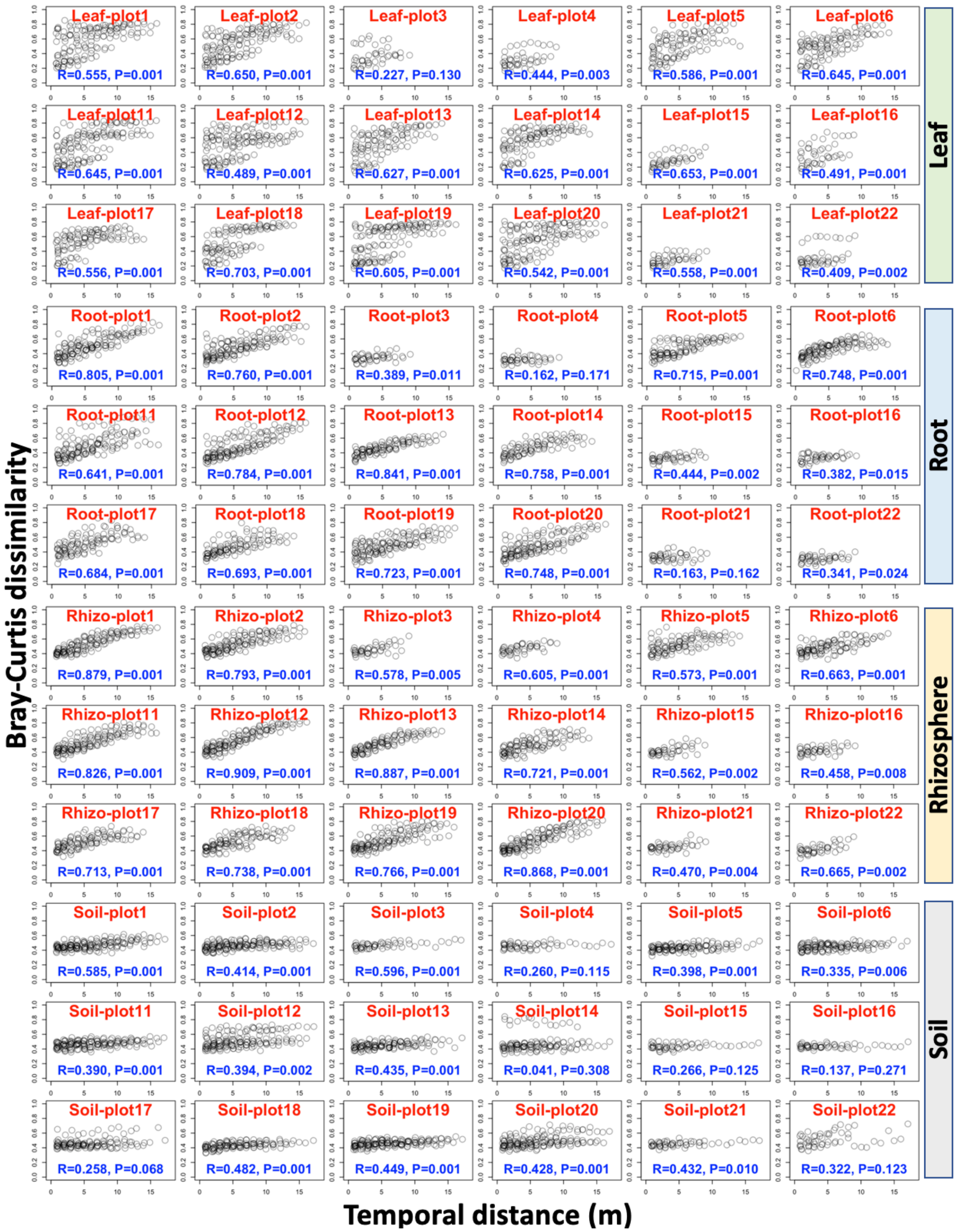

Supplementary Fig. 7 Succession of the fungal community by compartment and plot. Temporal distance (in weeks between sampling times) in relation to Bray-Curtis community dissimilarity by Mantel testing. Note the relatively large variance in Bray-Curtis community dissimilarity over small temporal distances in leaves. Source data are provided as a Source Data file.

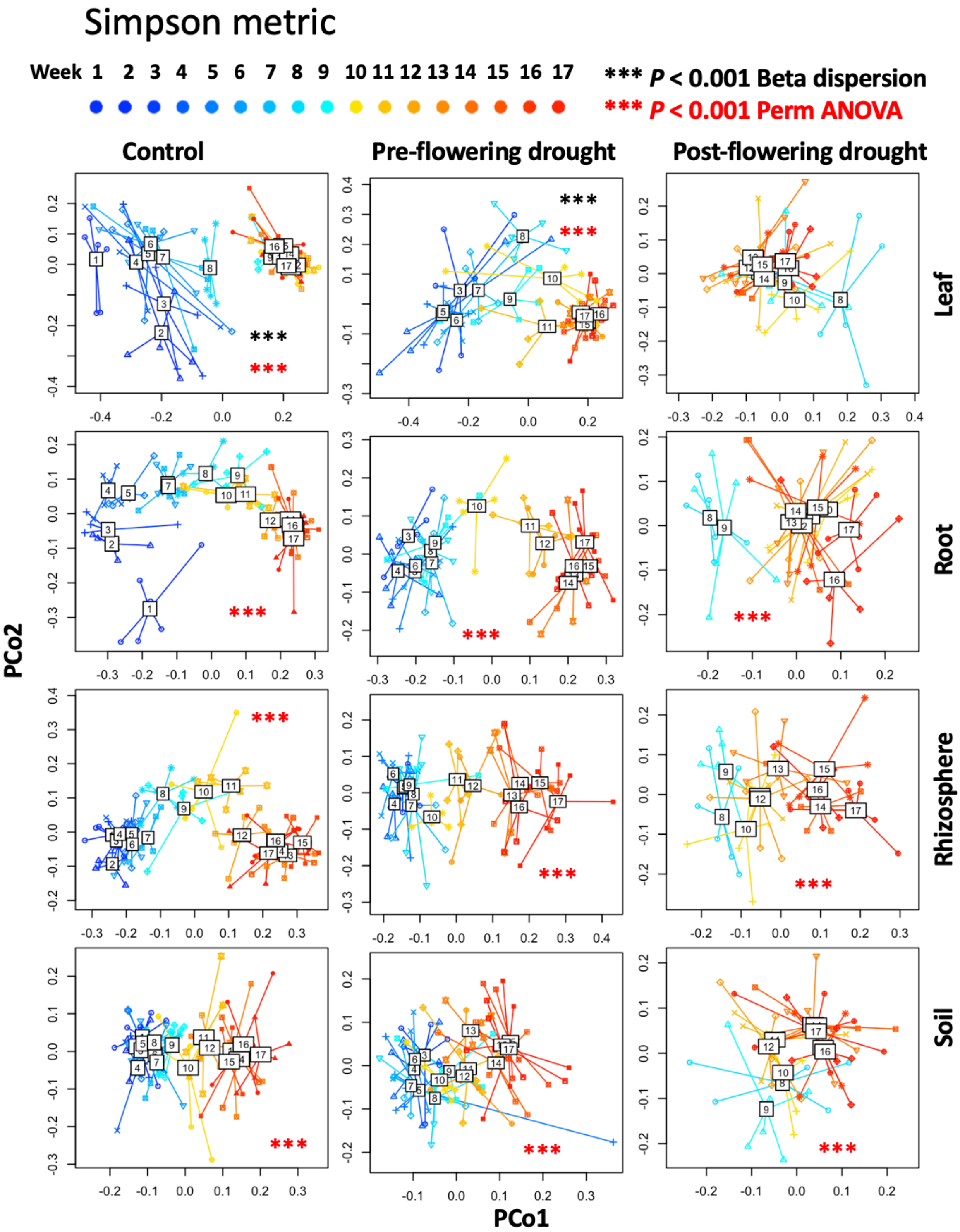

Supplementary Fig. 8 Temporal turnover of compositional variance. Principal coordinate (PCo) plots demonstrating the compositional variance as measured by Simpson metric within every week of three treatments and four compartments. ***$P < 0.001$ in beta dispersion analysis; *** $P < 0.001$ in permutational analysis of variance (Perm ANOVA). Note that the control results are also presented in Fig. 3B. Note the Simpson metric differs from the Bray-Curtis and Jaccard metrics in that it is free from richness variance. Source data are provided as a Source Data file.

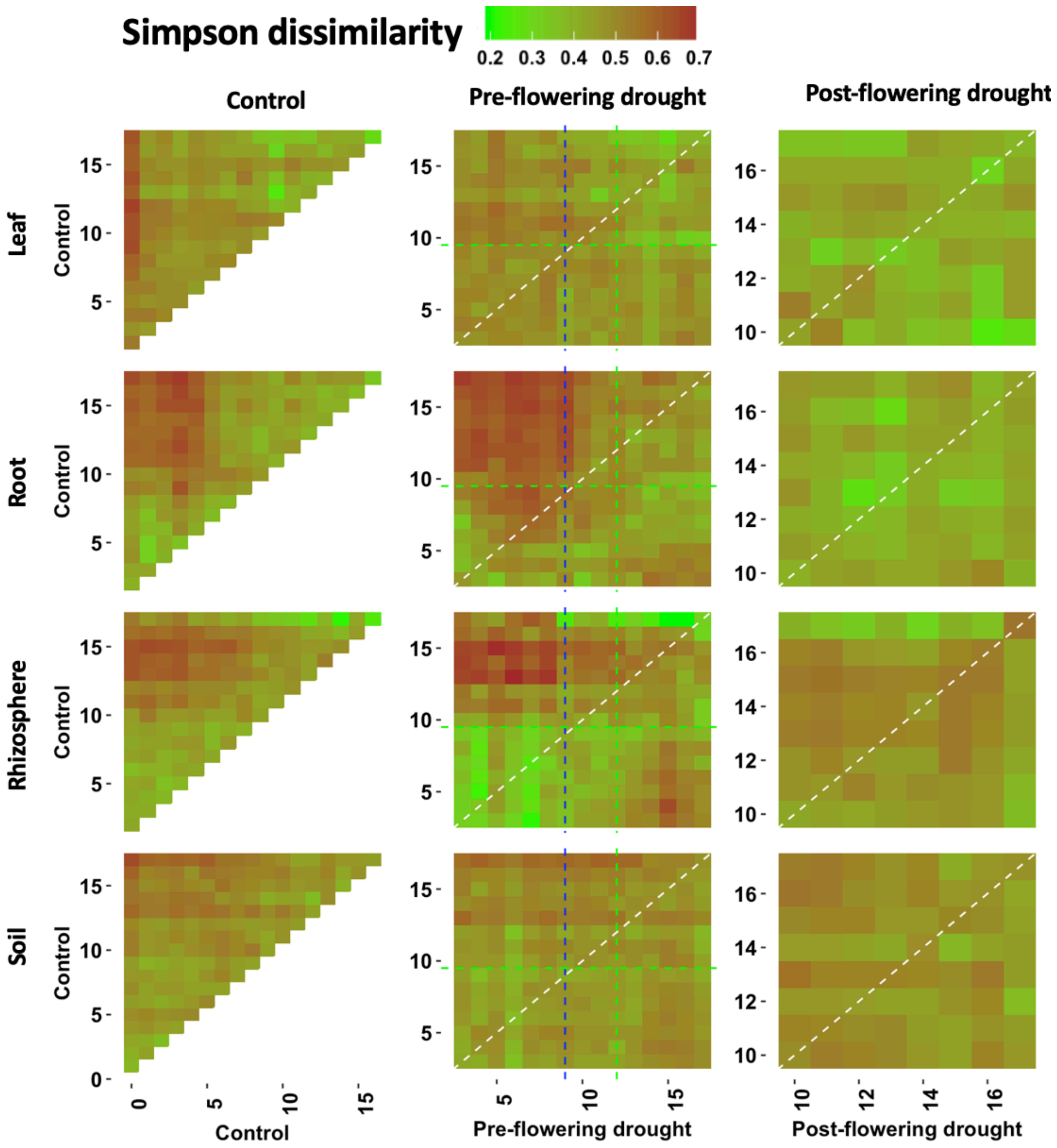

Supplementary Fig. 9 Temporal turnover of compositional variance between specified pairs of treatments. Heatmaps showing mean pairwise Simpson dissimilarity between time points between control and control, between control and pre-flowering drought, and between control and post-flowering drought. The largest turnover between sampling times is seen in leaves between the first week and subsequent time points, in roots between weeks 1-5 and weeks 11-17, and in the rhizosphere between weeks 1-7 and weeks 13-16; soils did not show significant turnover. The green dashed lines are flowering times. The blue dashed line is the time that pre-flowering drought was ended and water irrigation resumed. Source data are provided as a Source Data file.

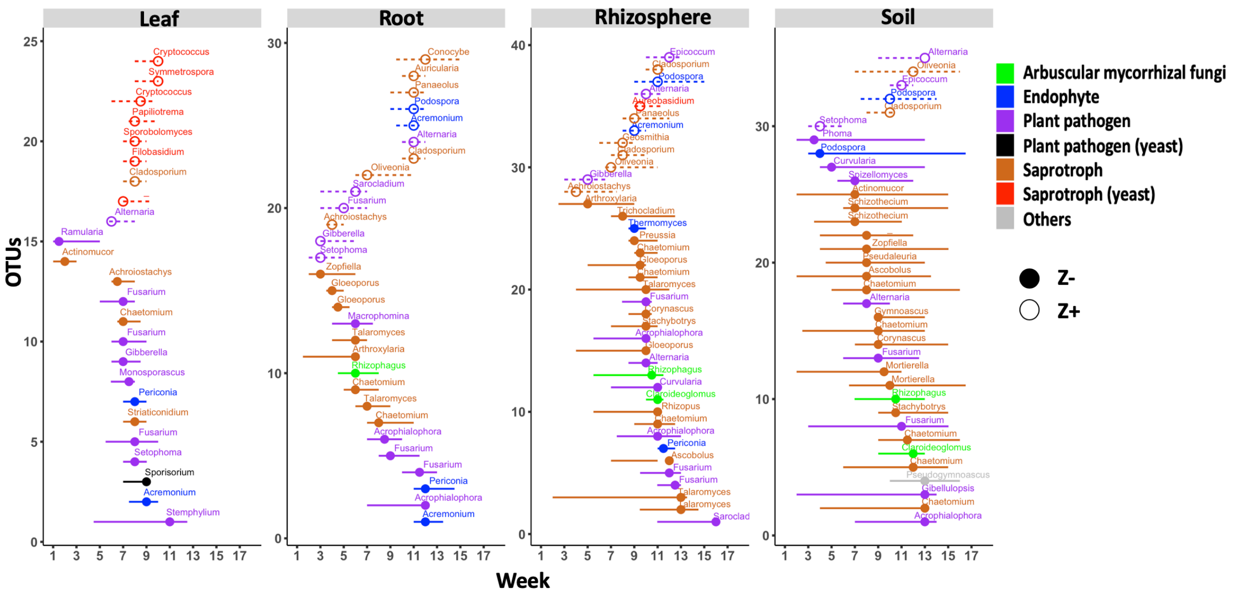

Supplementary Fig. 10 Threshold indicator taxa analyses (TITAN) showing individual OTU abundances in response to sampling time in the four compartments. This figure is the same as Fig. 3C, except for the addition of the genus names of z+ and z- OTUs. Source data are provided as a Source Data file.

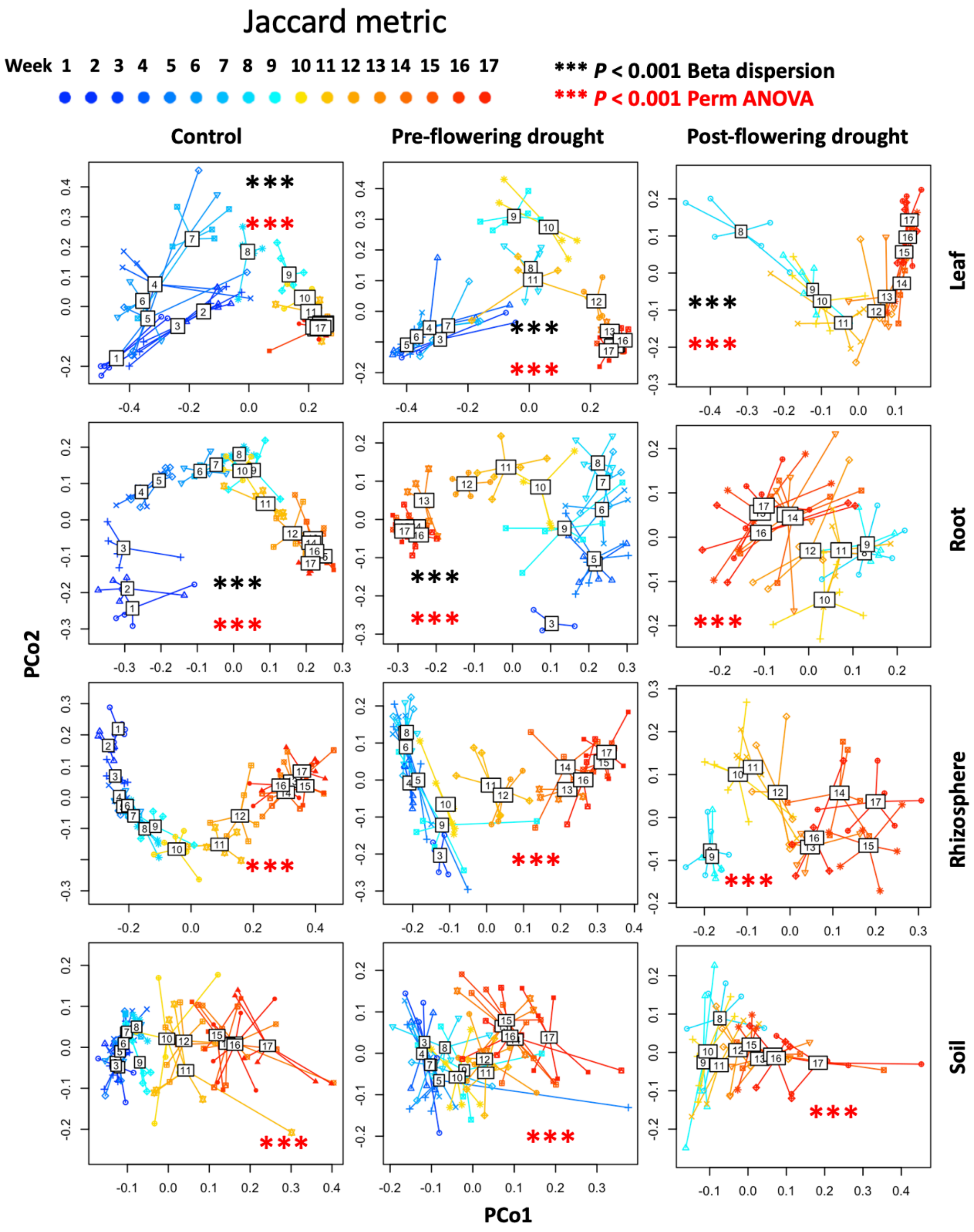

Supplementary Fig. 11 Principal coordinate (PCo) plots demonstrating the Jaccard dissimilarity within every week in three treatments and four compartments. Note the Jaccard metric is confounded by richness variance. ****P* < 0.001 in beta dispersion analysis; *** *P* < 0.001 in permutational analysis of variance (Perm ANOVA). Source data are provided as a Source Data file.

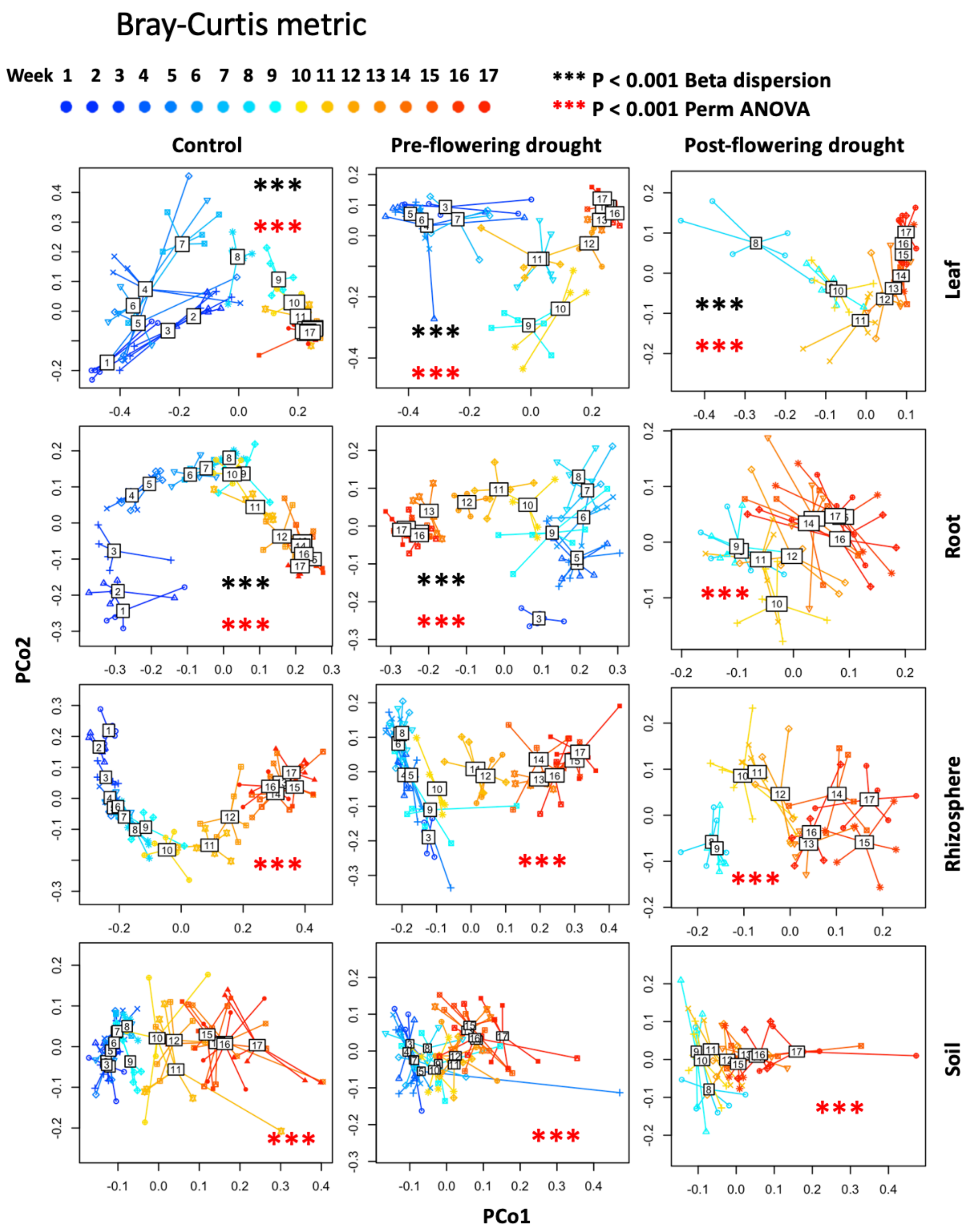

Supplementary Fig. 12 Principal coordinate (PCo) plots demonstrating the Bray-Curtis dissimilarity within every week in three treatments and four compartments. Note the Bray-Curtis metric is confounded by richness variance. ****P* < 0.001 in beta dispersion analysis; *** *P* < 0.001 in permutational analysis of variance (Perm ANOVA). Source data are provided as a Source Data file.

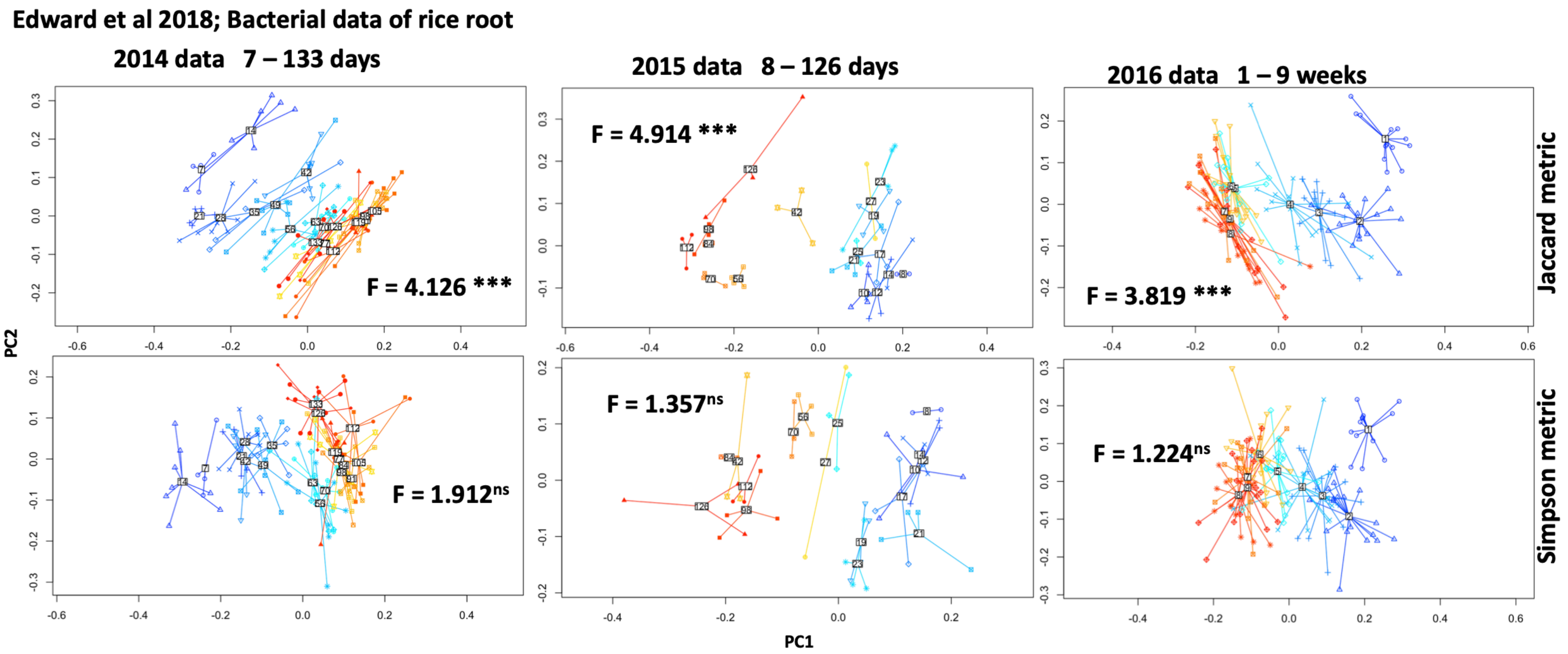

Supplementary Fig. 13 Re-analysis rice root bacteria data showing no beta dispersion of the turnover component of compositional variance. This result is found despite significantly higher compositional variance early in the rice life cycle as compared to later stages as measured by the Jaccard dissimilarity. *** $P_{adj} < 0.001$; ns: not significant; in beta dispersion analysis. Note the P values are adjusted by Bonferroni method to avoid type I error of multiple comparisons. Source data are provided as a Source Data file.

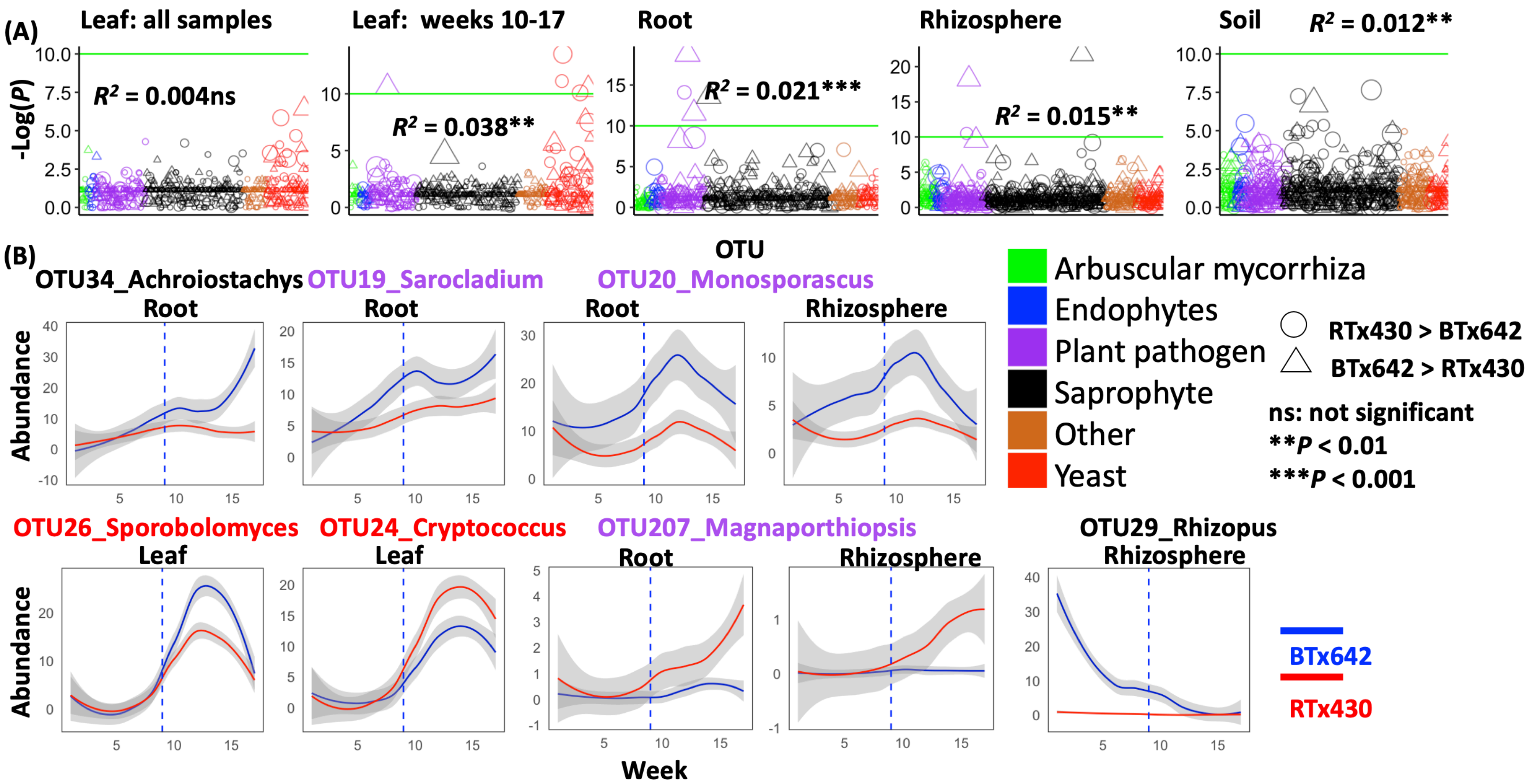

Supplementary Fig. 14 Sorghum cultivar effect on fungal OTU abundance and community composition in leaf, root, rhizosphere and soil. (A) Fungal OTUs showing significantly different abundance between sorghum cultivars BTx642 and RTx430. OTUs above a false discovery threshold [green horizontal line with $P < 0.00005$ (0.05/1070 OTUs) or -log (P) = 10] show significant bias between the two cultivars. The symbol size corresponds to OTU abundance (log transformed) and color corresponds to fungus functional guild. Note that cultivar effects were seen on OTU abundance in root and rhizosphere, but not in leaf nor soil. Differences in fungal community composition between the two sorghum cultivars, are given as R^2 as determined by permutational analysis of variance (PERM ANOVA). **(B) Divergent temporal abundance of Fungal OTUs with significant cultivar effects** (seen in A) can occur throughout sorghum development, e.g., at early (OTU29), late (OTUs 24, 26, 34, 207), or the middle time points (OTU19, 20). Source data are provided as a Source Data file.

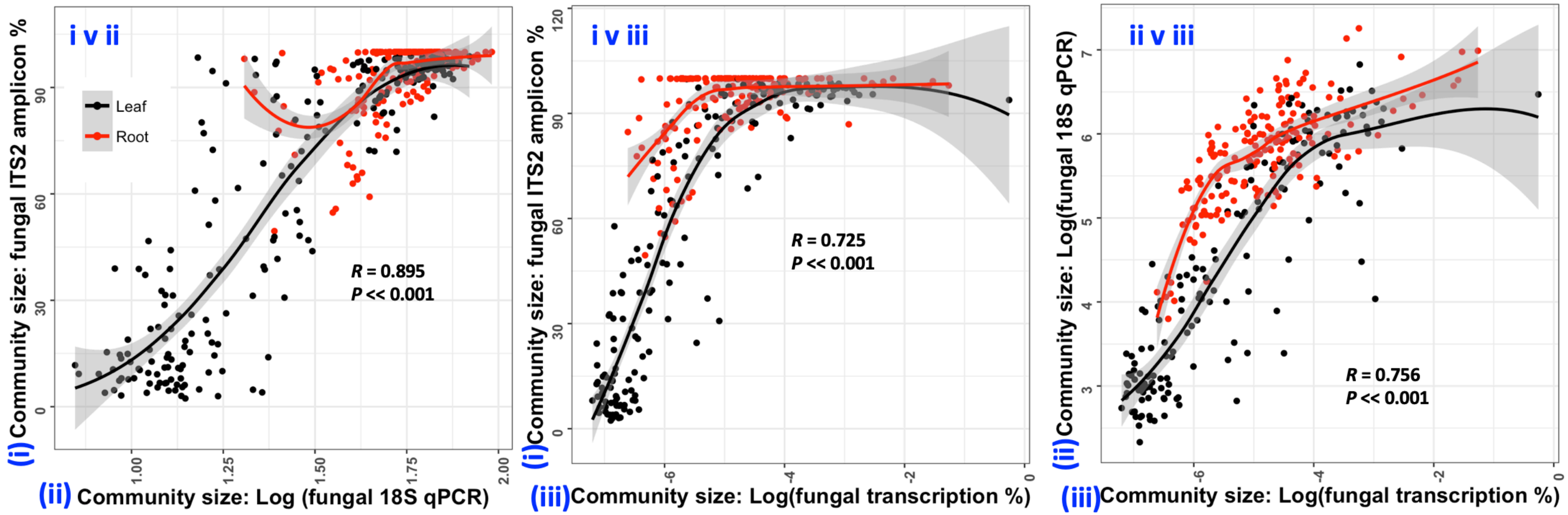

Supplementary Fig. 15 Comparison of three methods to evaluate fungal community size showing high consistency: (i) the percentage of fungal reads found in PCR amplifications of rDNA internal transcribed spacer 2 (ITS2) from fungal and host DNA, (ii) the fungal abundance as assessed by real time PCR amplification of rDNA small subunit (SSU, 18S) and (iii) the percentage of fungal reads found in the transcriptomes of sorghum leaves and roots. Source data are provided as a Source Data file.

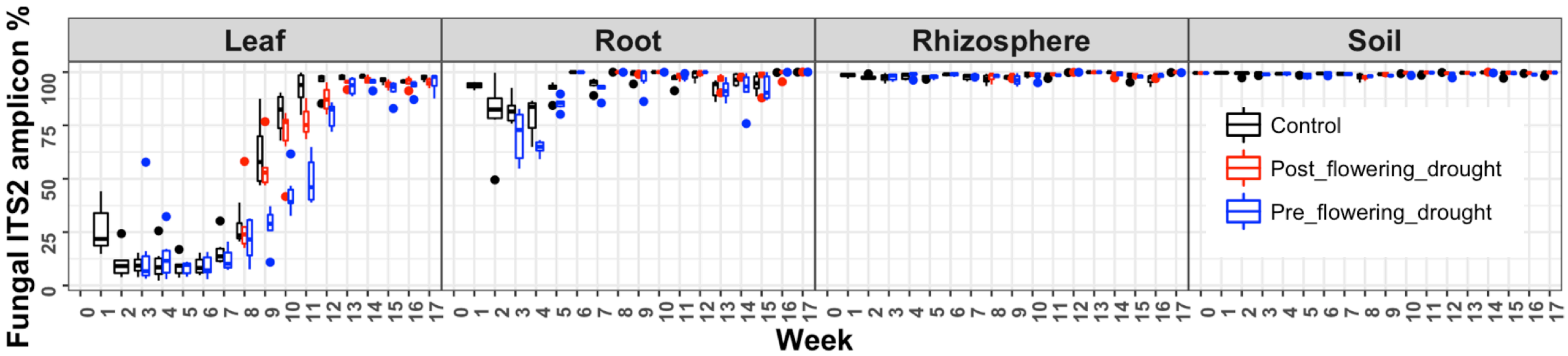

Supplementary Fig. 16 The temporal dynamics of the percentage of fungal reads compared to total plant and fungal reads in ITS2 amplicons in three treatments and four compartments. Source data are provided as a Source Data file.

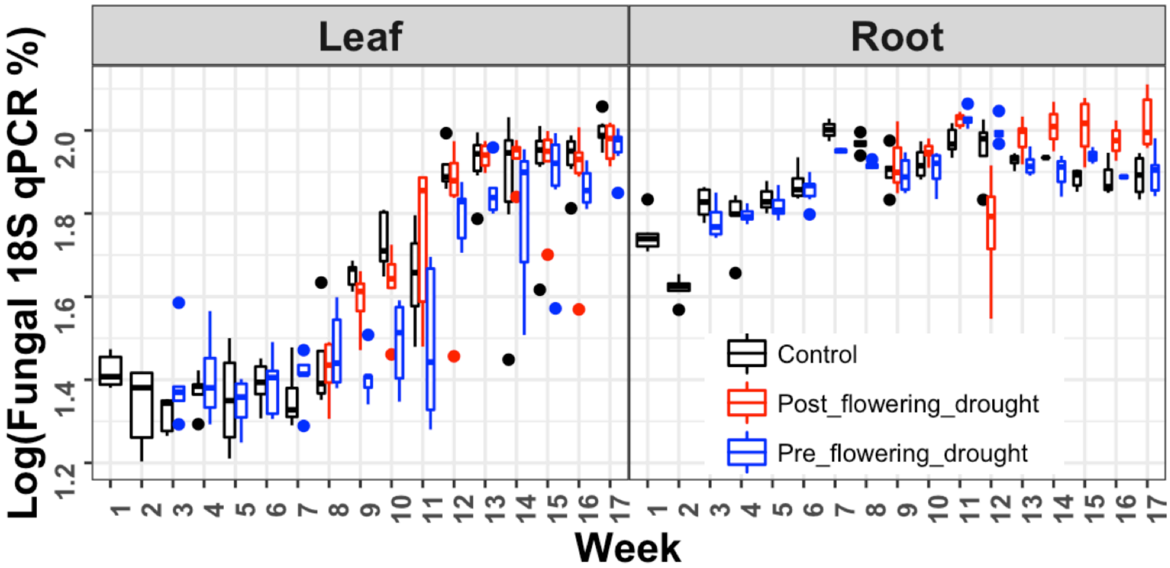

Supplementary Fig. 17 The temporal dynamics of the fungal abundance as detected by real time PCR of fungal 18S rDNA in three treatments of roots and leaves. Source data are provided as a Source Data file.

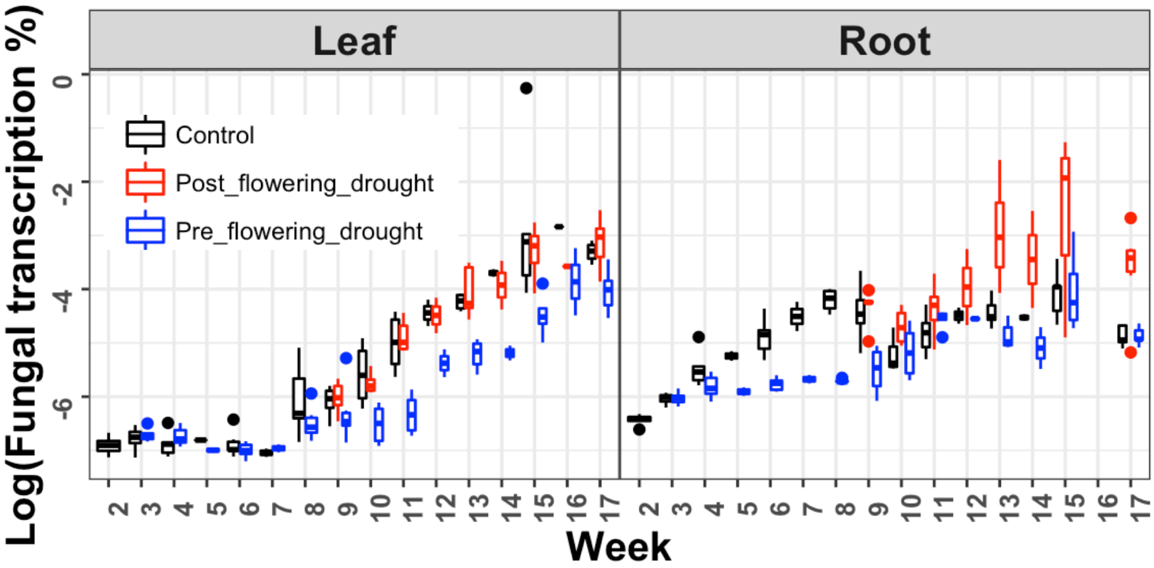

Supplementary Fig. 18 The temporal dynamics of the percentage (log transformed) of fungal reads compared to fungal and plant reads in sorghum transcriptome. Source data are provided as a Source Data file.

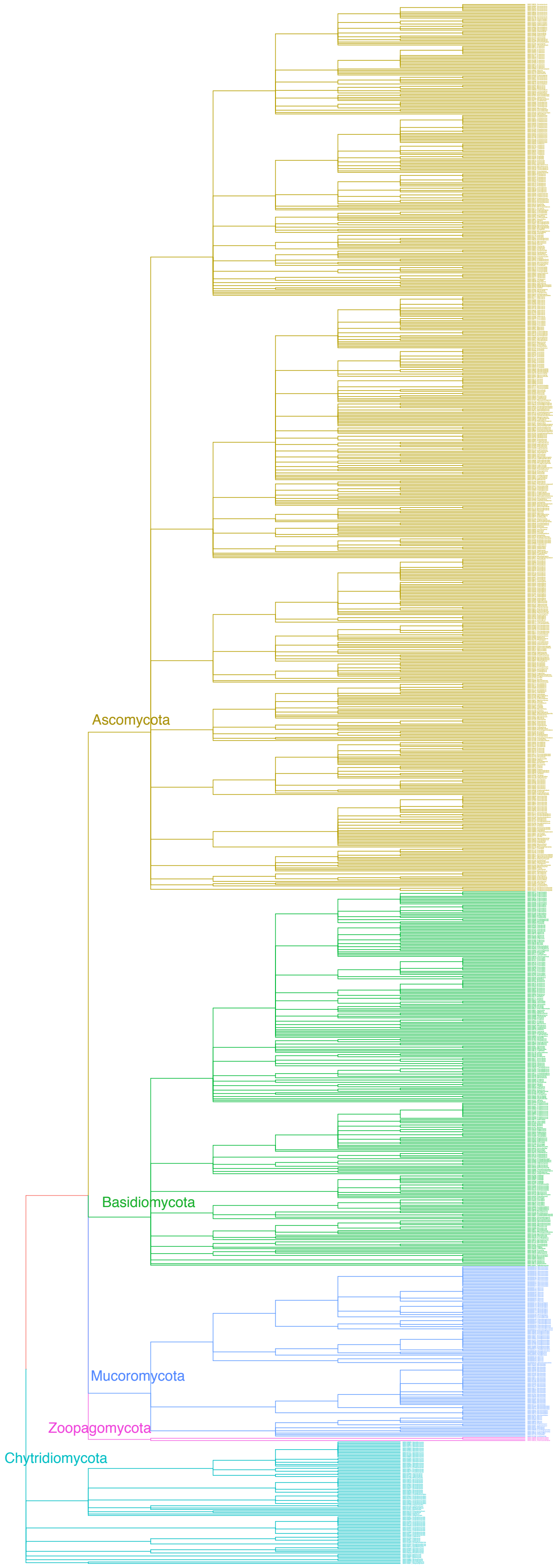

Supplementary Fig. 19 Fungal phylogeny based on 18S+28S rDNA sequence (taxonomy_to_tree.pl script of Tedersoo *et al* [31]) used to support βNTI analysis of 1070 fungal ITS2 OTUs obtained in this study. Source data are provided as a Source Data file.

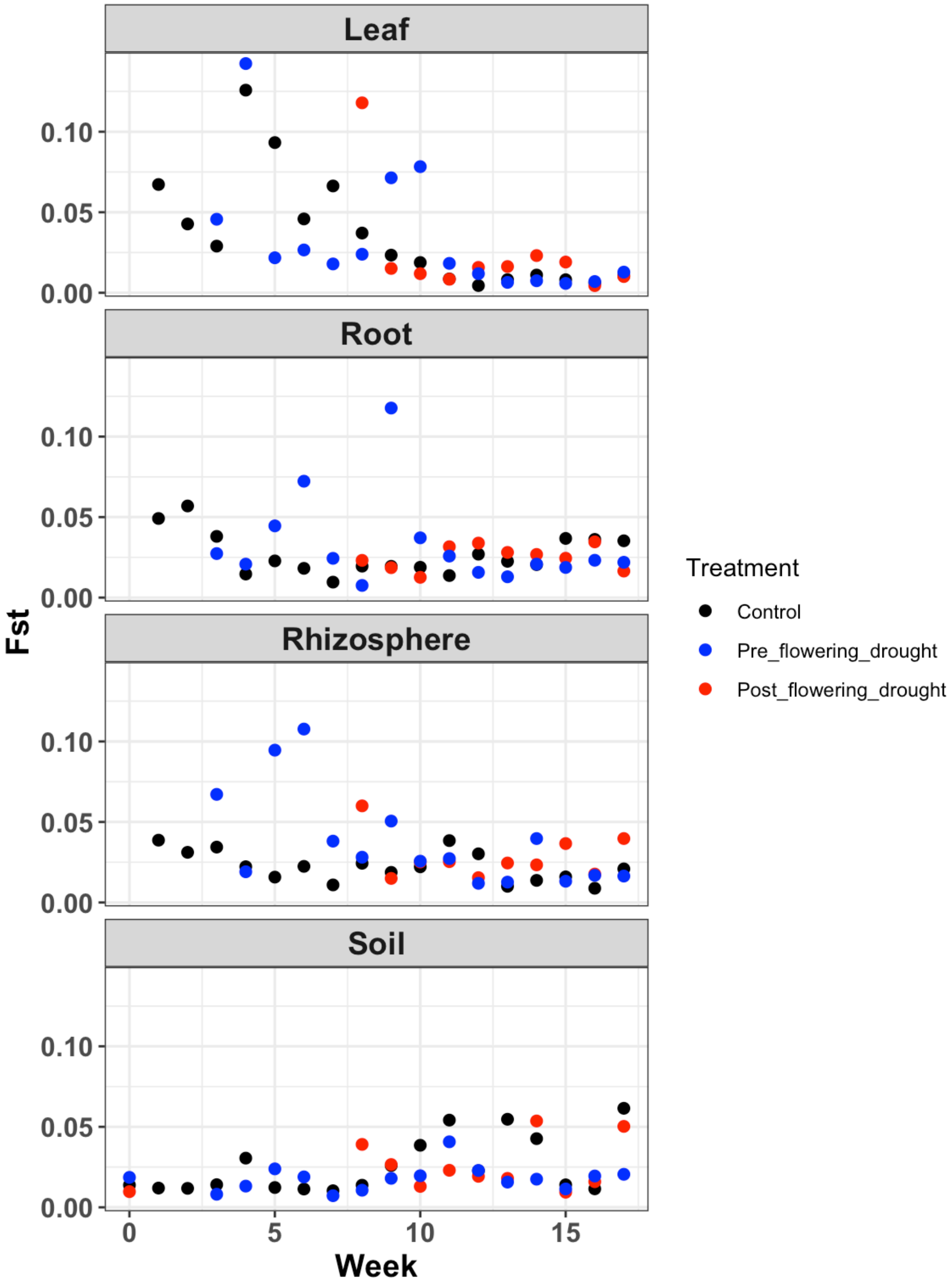

Supplementary Fig. 20 Temporal dynamics of fungal compositional variance measured by Fst. According to Gilbert and Levine [34], given the observed relative abundance, Fst is a measure that standardizes the variation among patches for each species relative to the maximum variation possible. Note due to the heteroskedasticity, curve fitting is not carried out for this dataset. Source data are provided as a Source Data file.

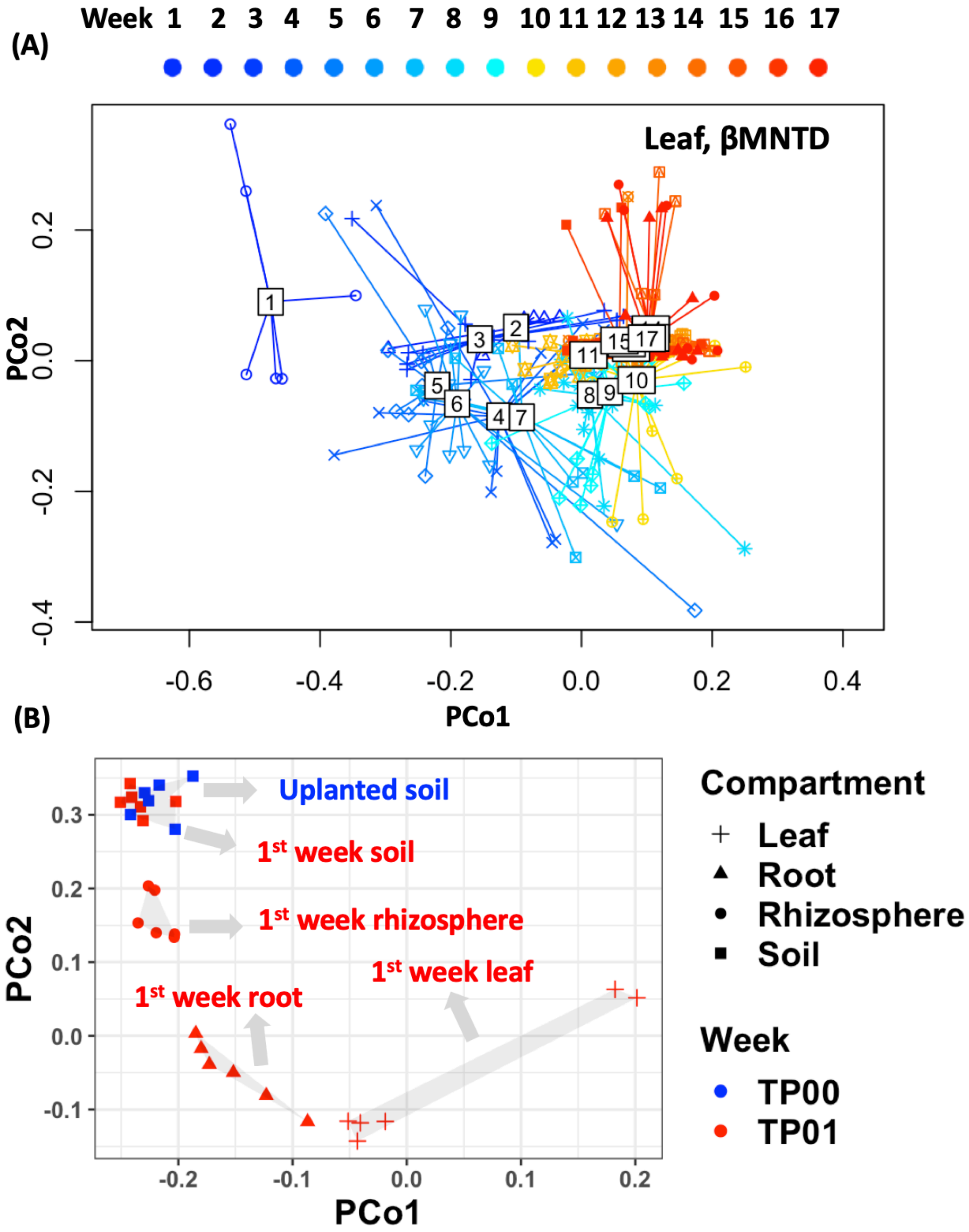

Supplementary Fig. 21 (A) Principal coordinate (PCo) plots of beta mean nearest taxon distanc (βMNTD) for fungal communities. The first week leaf samples are phylogenetically distinct from other samples, as demonstrated by PCo analysis of βNTI of fungal communities. **(B) PCo plots of fungal communities in unplanted soil and first week samples.** Replicate communities of the soil and rhizosphere for these first two time points (TP00 and TP01) are more similar than are the communities of leaves or roots for just the second time point. Source data are provided as a Source Data file.

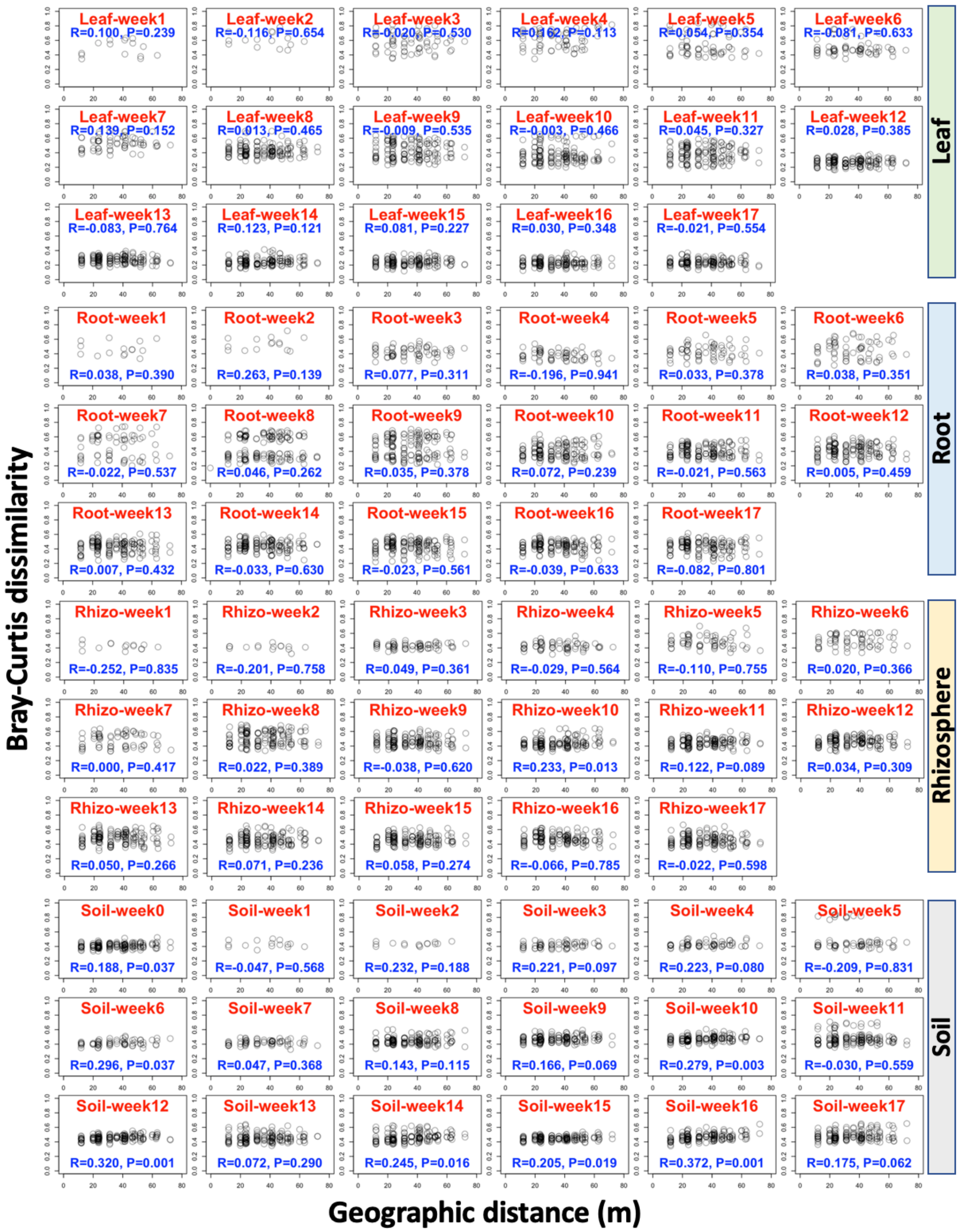

Supplementary Fig. 22 Evidence of the absence of distance decay of fungal community dissimilarity, as seen from the nearly flat slope of the correlation between geographic distance and Bray-Curtis dissimilarity of fungal communities of each week and each compartment. Overall, the null hypothesis of no relationship between geographic distance and community dissimilarity, while taking into account multiple testing [P = 0.003 (0.05/17)], cannot be rejected for any leaf, root or rhizosphere sample, or for 15 of 17 soil samples (excepting weeks 12 and 16). Thus, although there is an association at two time points between community dissimilarity and distance for post-flowering soil, the R is low (0.320-0.372) and this effect is seen weeks after our detection of stochasticity in leaves and roots. Source data are provided as a Source Data file.

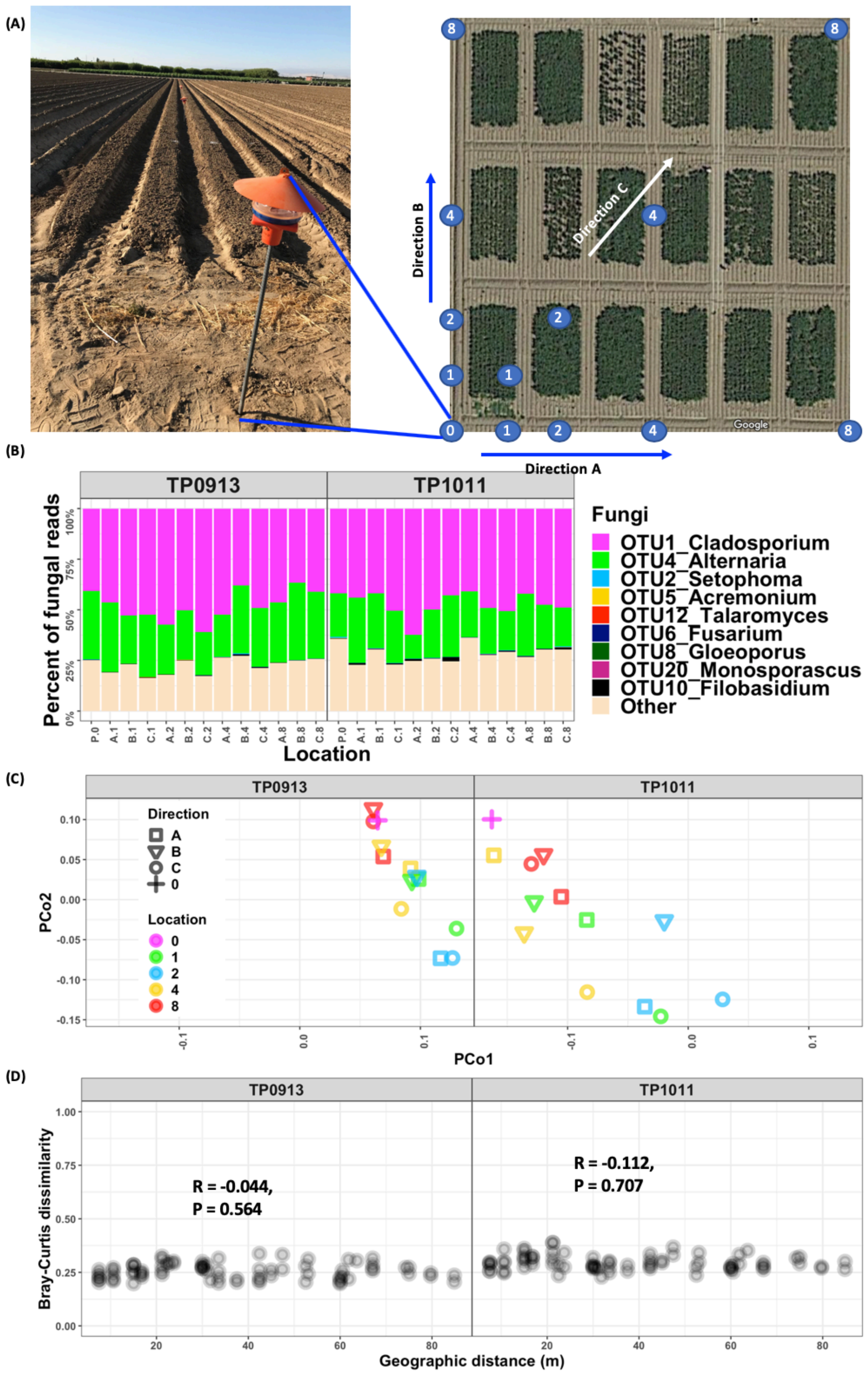

Supplementary Fig. 23 Evidence of the absence of distance decay of air fungal community dissimilarity. (A) air samplers were located in the sorghum field with a nested design. (B) Air fungal community composition in each sample. (C) Principal coordinate (PCo) analysis of air fungal community in terms of direction and location of samplers. (D) Flat relationship between geographic distance and Bray-Curtis dissimilarity of air fungal communities. Source data are provided as a Source Data file.

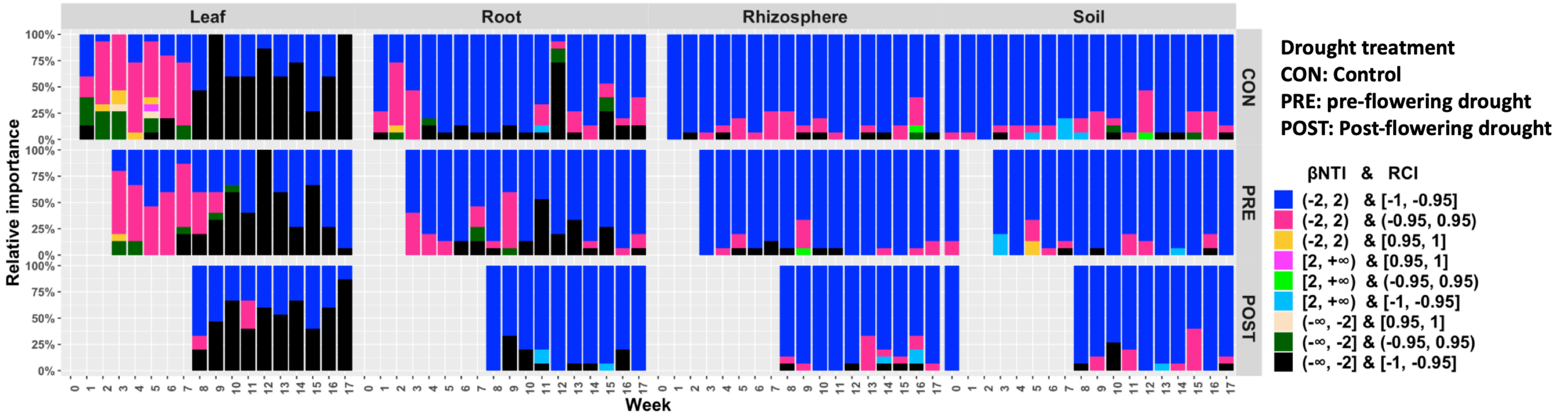

Supplementary Fig. 24 The percent of fungal compositional variance governed by various processes in every week in control, pre- and post-flowering drought in leaves, roots, rhizospheres, and soils. Source data are provided as a Source Data file.

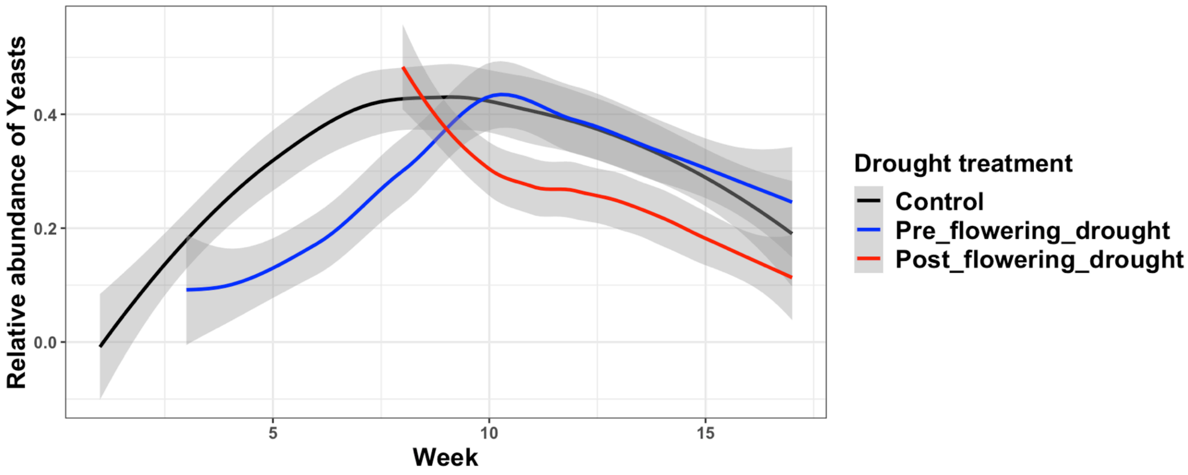

Supplementary Fig. 25 The temporal dynamics of yeasts on leaves in control, pre- and post-flowering droughts. Source data are provided as a Source Data file.

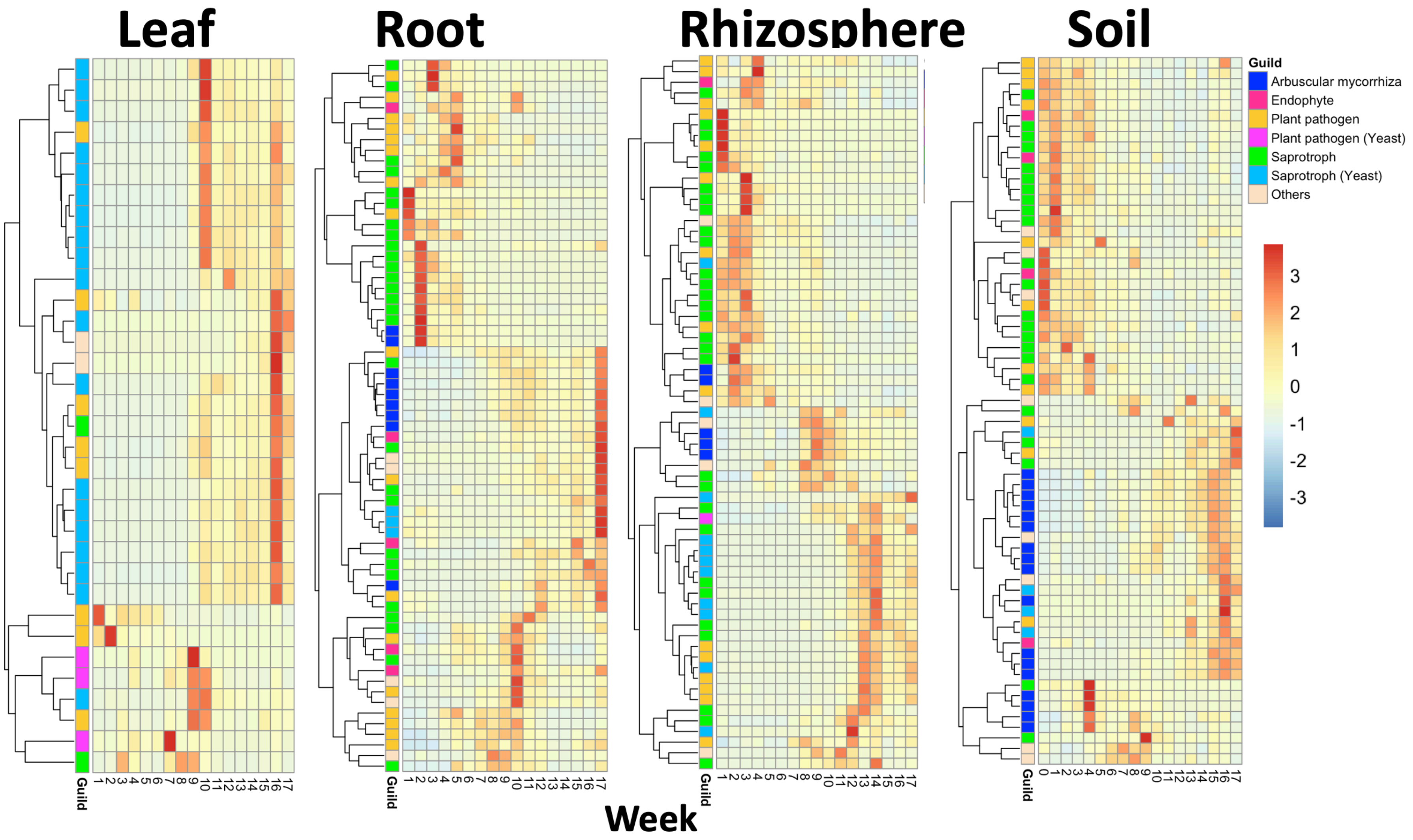

Supplementary Fig. 26 Heatmap showing the abundance of fungal OTUs that are predictive of sampling time as determined by random forest analysis. Source data are provided as a Source Data file.

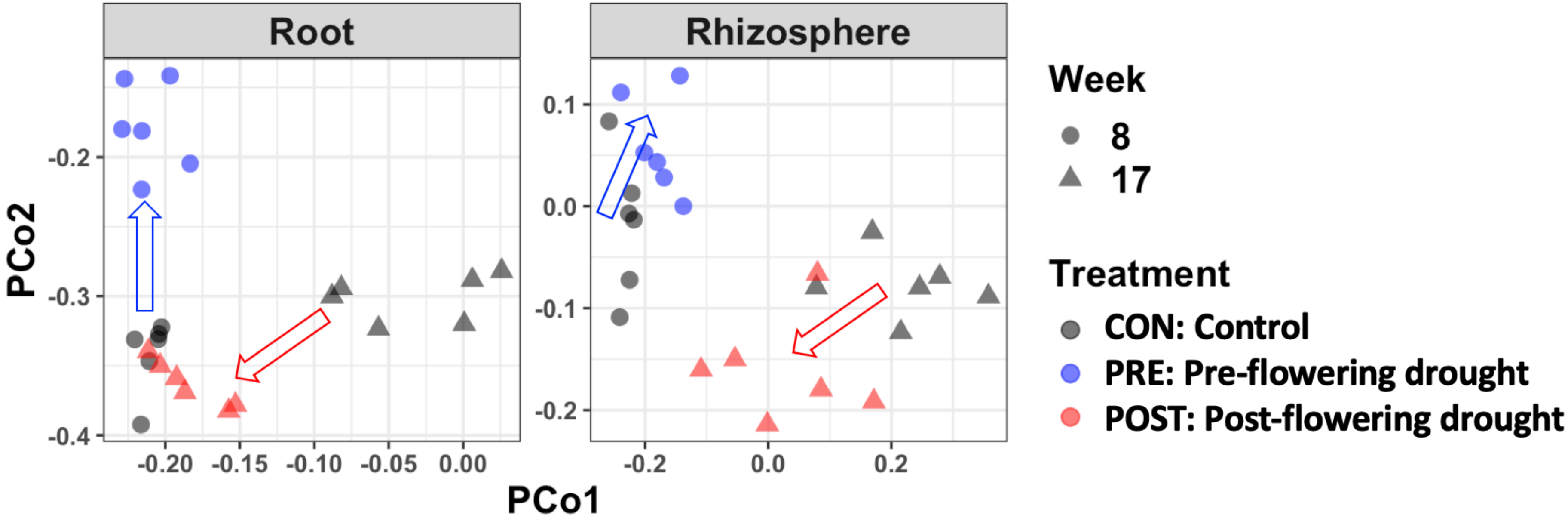

Supplementary Fig. 27 Effect of pre and post- flowering drought on fungal community composition in root and rhizosphere. Note the week 8 and week 17 are differentiated by circle and triangle shapes, and the three treatments are distinguished by colors. The blue and red arrows show fungal community changes caused by pre- and post- flowering droughts. Source data are provided as a Source Data file.

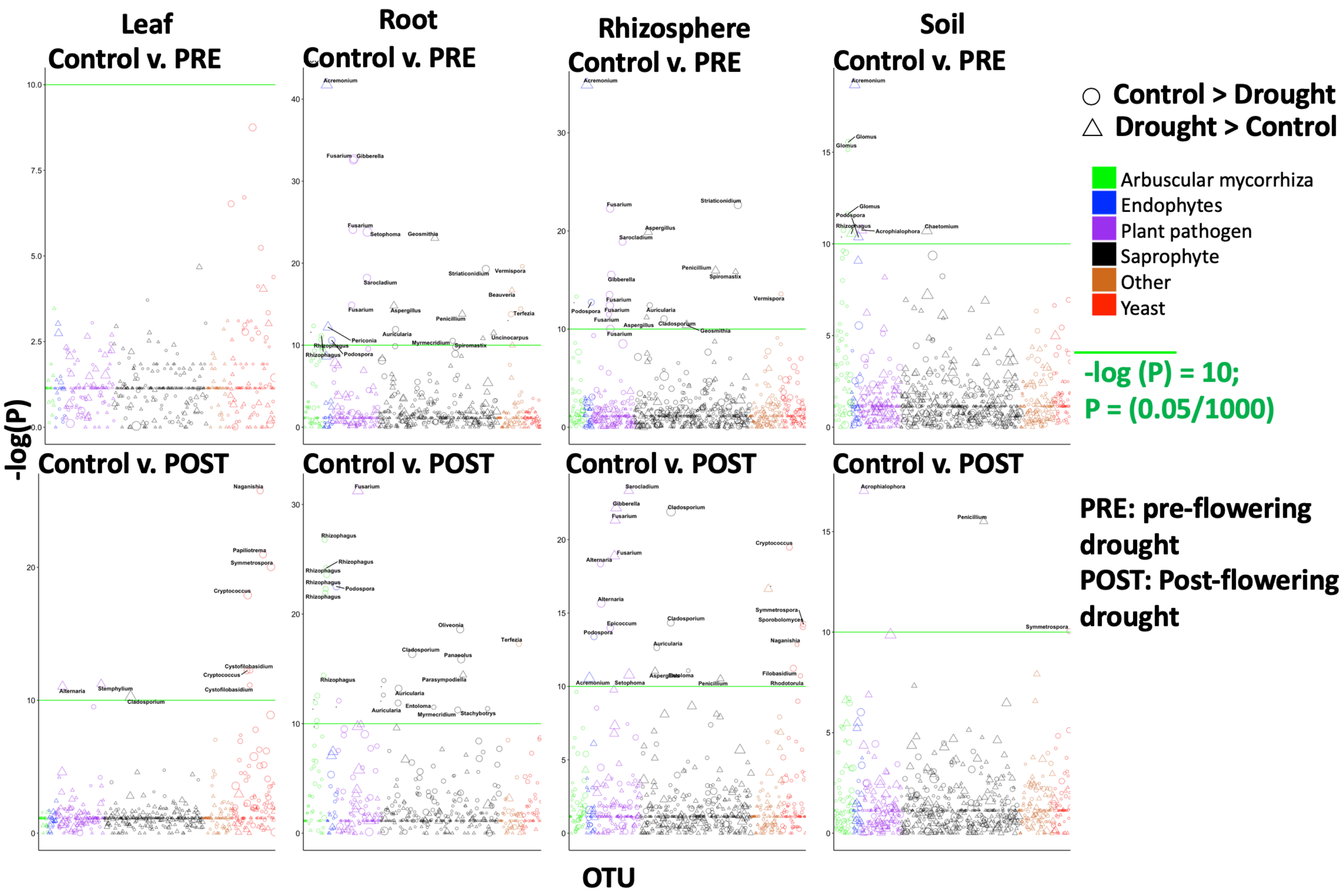

Supplementary Fig. 28 OTU abundance and drought. Manhattan plot showing fungal OTUs significantly differently between control and pre-flowering drought and between control and post-flowering drought. OTUs above the green horizontal line [-log (P) = 10] are significant biased fungi detected by $P < 0.00005$. The symbol size corresponds to OTU abundance (log transformed). This figure is the same as Fig. 4A-B, except that genus names are provided for significantly different OTUs. Source data are provided as a Source Data file.

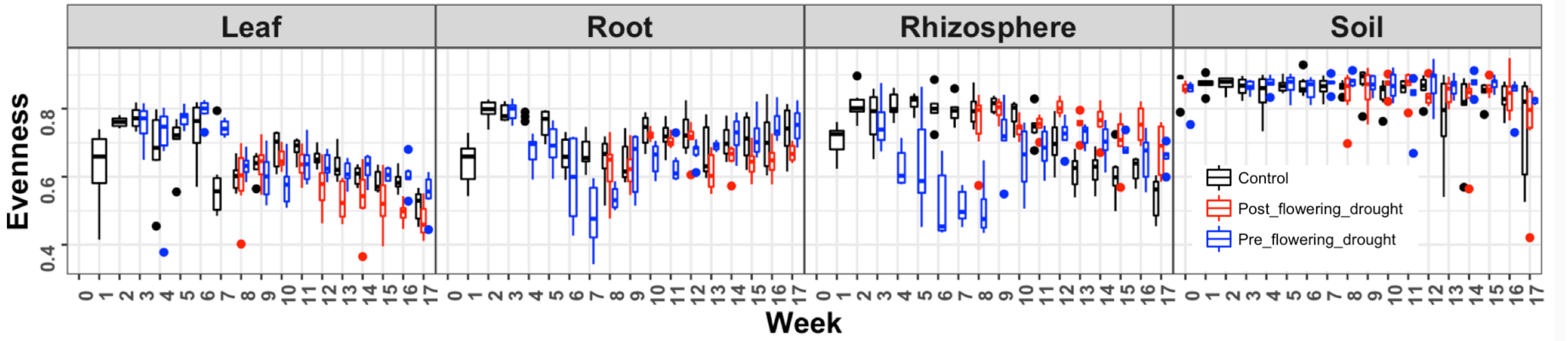

Supplementary Fig. 29 Fungal community evenness by week in leaves, roots, rhizospheres and soils. Source data are provided as a Source Data file.
