# Supplementary material for: Fungal community assembly in drought-stressed sorghum shows stochasticity, selection, and universal ecological dynamics
Source: Nat Commun. 2020 Jan 7;11:34. doi: 10.1038/s41467-019-13913-9 (PMC6946711; doi:10.1038/s41467-019-13913-9)
Supplement: Supplementary file 3 — Description of Additional Supplementary Files [file 41467_2019_13913_MOESM3_ESM.pdf]

Description of Additional Supplementary Files

File Name: Supplementary Data 1

Description: A list of studies investigating the fungal communities at host plant dimensions of time, compartment and genotype, as well as changing watering regime

File Name: Supplementary Data 2

Description: A list of recent studies of bacterial microbiome at host dimensions of time, compartment and genotype, as well as changing watering regime

File Name: Supplementary Data 3

Description: Fungal identification in this study

File Name: Supplementary Data 4

Description: Permutational analysis of variance (PERM ANOVA) showing association of fungal community composition with compartment, time point, drought treatment, sorghum cultivar and their interactions

File Name: Supplementary Software 1

Description: The interactive Krona figures of fungi in unplanted soil and sorghum Mycobiome
