# Supplementary material for: Fungal community assembly in drought-stressed sorghum shows stochasticity, selection, and universal ecological dynamics
Source: Nat Commun. 2020 Jan 7;11:34. doi: 10.1038/s41467-019-13913-9 (PMC6946711; doi:10.1038/s41467-019-13913-9)
Supplement: Supplementary file 8 — Supplementary Software 1 [file 41467_2019_13913_MOESM8_ESM.zip › Supplementary Software1/Supplementary Software1/Krona of fungi in unplanted soil.html]

Javascript must be enabled to view this page.

magnitude
magnitudeUnassigned

S0.guild

1159492

29075

0

0

3070

0

136

0

4557

8112

4

0

80

7

29

64

150

8

57

0

0

186

88

654

58

0

2

42

1

0

0

20

0

602

0

0

3

9

9

0

32

567

53

1834

103

0

5577

4

0

8

1364

1113

447

3

0

0

0

0

0

22

204

7

9

65

42

81

0

0

0

0

660663

1

0

39644

26997

8690

10

0

0

144

677

5

99

70

5891

23

36415

947

17

6

38

3680

0

1

1279

0

6613

14

0

35686

20951

69

26

21861

0

7

6057

1882

24

0

0

1850

5719

16

0

222

4

0

0

0

9

93

81

2783

30

2596

21

35337

0

10

0

1006

191

50

26

465

7

2033

97

2456

40

0

256

0

1961

508

34

2

246

8

0

0

47

1

0

177

10673

1016

7

4308

2929

0

217

379

28355

0

0

4

21

118

14

83

9

139

1

1478

50

2653

1180

0

3

75

7

5722

0

175116

0

1

13520

236

1470

0

58

8908

6932

937

5

71

12307

0

52

159

9

138

13

249

2

0

219

0

5

0

13208

0

5

346

603

128

0

0

121

7

323

1

0

0

3

23

5

61731

6

3

4

1993

709

2772

565

0

5254

7867

0

4

94

513

8

139

3

13

0

0

853

7

79

374

0

83

0

54

493

1

19

38

5351

0

33

10

62

0

1

574

126

12545

0

0

34

0

13

0

7

0

3

17

0

28

0

0

0

0

4

0

0

0

0

1

0

0

5

0

4

0

0

6

2

0

0

121

0

0

220

12

9810

1

10

777

76

0

29

0

106

0

1

71

0

0

0

108

505

574

0

68543

0

0

20588

15136

4793

24

6663

68

21271

0

388462

83

3

1095

2019

3500

0

12571

0

6525

70

0

1996

16238

1214

144

0

37

91

37

1068

180

0

1517

139

9

0

3

66863

8

0

372

25274

0

0

0

13

232

0

0

5692

4

43

12

18994

16

124

32

1351

21273

567

10

1

104

30

6

11

7

2238

0

426

0

0

0

185126

2648

4706

0

0

26

1628

52

0

9

0

1

181

498

28

23

0

0

1148

145

1
