# Supplementary material for: Fungal community assembly in drought-stressed sorghum shows stochasticity, selection, and universal ecological dynamics
Source: Nat Commun. 2020 Jan 7;11:34. doi: 10.1038/s41467-019-13913-9 (PMC6946711; doi:10.1038/s41467-019-13913-9)
Supplement: Supplementary file 8 — Supplementary Software 1 [file 41467_2019_13913_MOESM8_ESM.zip › Supplementary Software1/Supplementary Software1/Krona of sorghum Mycobiome.html]

Javascript must be enabled to view this page.

magnitude
magnitudeUnassigned

Sorghum.guild

33382266

2604686

6859

44601

8

263

139748

23198

347

362330

752

542

703

72

89

10185

141

60310

435

27

737

2017

140

69

180

79

202

53699

129

34

402

4641

24

353389

565019

287

25052

111

546

308294

52

1155

931

321241

344

1487

839

310

24518

55

1117

76

269967

85

3185

13125

242

57

239

0

0

12792977

30

2431327

1182520

512

992

1374

2952931

84564

408872

22

31

8914

61308

7

19389

1858

183

12958

600

394

12698

28790

2183

14487

60

107

517

2864

109807

17

413

21224

50

50

11876

3

505

63300

4046

350

38

623

207

32

293

640

16909

462

32

789

28596

698

398

48750

23022

163

58167

306042

312

6

24

164

106070

16661

84

2513

352

8360

5074

97

316

401

3423698

818043

470959

79

225

2270

7766

988

313

105

36

67

328309

954

179

168

39

98

496

39

543

40838

6468

192

25

106

181

179

19

18

43

29

573

90

93

6376

34

932

117

48

2015

499

48

31967

33

7838

3985

28637

84

59

4

125936

3588

433

12

43

1189

18208

3

25318

2891

19

8403

25

17

698

53

236

677

4612

1902

386995

18609

119

219668

143

85872

14752

47832

9419577

270658

142

112782

11

55730

24

358271

150

5879728

602

2741479

7849722

1573262

1015

342722

206

33338

53480

303

156

22545

497243

613

662

935

2

0

9854

1022

435

219

710

8600

1196

100

979

112

1986

105639

296

39

39

153

15743

195301

148

82

99243

13045

19324

5038

12180

27255

152

109

1260

956

46628

14514

16

166152

83

160

304

754242

54366

15385

57478

44

157

306

2998

438

1540

162

578

2982

29

51

331490

18

8230

22

302

20684

123

7

10363

138124

350

20929

955

64220

34

187475

1958

11240

790

116

16382

61

1950

5799

45

200

1149

15

197

25897

1048

50

34

3568

165

96

37783

889

21296

41

85

4173

101

19959

3178

81641

1766

90065

13

1273

12

723

181

30

1441

174

169676

236

254

1213

64

1026

62

206

146

77649

13

1074

198

43

1121

439

146

318416

22

1003

167

183882

58

223

264

714

350

3734

238687

120

16690

108392

35

23

25594

72

2481

1083

2977

143

7939

411

62

4738

1207

45246

601

1773

264783

1017

17513

38631

1868

733231

497

8135

1076

125

835

33256

19077

2160

769

342

72

7651

3169

421

8084

32442

36314

1099

58141

55642

598

16881
