# Supplementary material for: Fungal community assembly in drought-stressed sorghum shows stochasticity, selection, and universal ecological dynamics
Source: Nat Commun. 2020 Jan 7;11:34. doi: 10.1038/s41467-019-13913-9 (PMC6946711; doi:10.1038/s41467-019-13913-9)
Supplement: Supplementary file 9 — Reporting Summary [file 41467_2019_13913_MOESM9_ESM.pdf]

nature research

Corresponding author(s): John W. Taylor

Last updated by author(s): Nov 11, 2019

Reporting Summary

Nature Research wishes to improve the reproducibility of the work that we publish. This form provides structure for consistency and transparency in reporting. For further information on Nature Research policies, see Authors & Referees and the Editorial Policy Checklist.

Statistics

For all statistical analyses, confirm that the following items are present in the figure legend, table legend, main text, or Methods section.

n/a	Confirmed	
☐	☒	The exact sample size (*n*) for each experimental group/condition, given as a discrete number and unit of measurement
☐	☒	A statement on whether measurements were taken from distinct samples or whether the same sample was measured repeatedly
☐	☒	The statistical test(s) used AND whether they are one- or two-sided<br>*Only common tests should be described solely by name; describe more complex techniques in the Methods section.*
☒	☐	A description of all covariates tested
☐	☒	A description of any assumptions or corrections, such as tests of normality and adjustment for multiple comparisons
☐	☒	A full description of the statistical parameters including central tendency (e.g. means) or other basic estimates (e.g. regression coefficient) AND variation (e.g. standard deviation) or associated estimates of uncertainty (e.g. confidence intervals)
☐	☒	For null hypothesis testing, the test statistic (e.g. *F*, *t*, *r*) with confidence intervals, effect sizes, degrees of freedom and *P* value noted<br>*Give P values as exact values whenever suitable.*
☒	☐	For Bayesian analysis, information on the choice of priors and Markov chain Monte Carlo settings
☒	☐	For hierarchical and complex designs, identification of the appropriate level for tests and full reporting of outcomes
☒	☐	Estimates of effect sizes (e.g. Cohen's *d*, Pearson's *r*), indicating how they were calculated

Our web collection on statistics for biologists contains articles on many of the points above.

Software and code

Policy information about availability of computer code

Data collection	FastQC v0.11.5, cutadapter v1.9.1 , USEARCH v8.0 , BLAST, FUNGuild v1.1
Data analysis	KronaTool v2.7, R packages: ggtern, TITAN2, ggplot2, RandomForest, CoDaSeq, vegan, Ape, base, betapart, lme4, MuMIn, DOC, igraph

For manuscripts utilizing custom algorithms or software that are central to the research but not yet described in published literature, software must be made available to editors/reviewers. We strongly encourage code deposition in a community repository (e.g. GitHub). See the Nature Research guidelines for submitting code & software for further information.

Data

Policy information about availability of data

All manuscripts must include a data availability statement. This statement should provide the following information, where applicable:

- Accession codes, unique identifiers, or web links for publicly available datasets
- A list of figures that have associated raw data
- A description of any restrictions on data availability

All data that support the findings of this study have been deposited in GenBank (representative read set) with the accession codes: No. MG008508 – MG008559, and MK018174 – MK019191; or Sequence Read Archive (raw data) with the accession codes: Bioprojects PRJNA412410 and PRJNA494573; Biosamples SAMN07711256 – SAMN07711567, SAMN10176611 – SAMN10176624, SAMN10173923 – SAMN10174030, SAMN10173711 – SAMN10173818, SAMN10173573 – SAMN10173680, SAMN10173450 – SAMN10173557, SAMN10173164 – SAMN10173298, SAMN10173035 – SAMN10173160, and SAMN10172702 – SAMN10172707. All scripts used in this study is available at GitHub (https://github.com/ChengGaoBerkeley/EPICON.Mycobiome).

Field-specific reporting

Please select the one below that is the best fit for your research. If you are not sure, read the appropriate sections before making your selection.

☐ Life sciences ☐ Behavioural & social sciences ☒ Ecological, evolutionary & environmental sciences

For a reference copy of the document with all sections, see nature.com/documents/nr-reporting-summary-flat.pdf

Ecological, evolutionary & environmental sciences study design

All studies must disclose on these points even when the disclosure is negative.

Study description	We investigated the Mycobiome of 1026 sorghum samples consisting four compartment, 17 time points, three treatments and two cultivars.
Research sample	We collected 1026 sample from three treatments, two sorghum cultivars, 17 time points and four compartments.<br>Three treatments: control, pre-flowering drought and post-flowering drought; Two sorghum cultivars: the pre-flowering, drought tolerant sorghum cultivar RTx430, and the post-flowering, drought tolerant (or 'stay green') cultivar BTx642; Four compartments: Leaf, Root, Rhizosphere, and Soil
Sampling strategy	Between 10:00 and 14:00 of every sampling date, at least ten individual sorghum plants were removed from randomly chosen locations within one of the central eight rows in each plot. To sample leaves, the 3rd and 4th youngest, fully expanded leaves of the ten plants were removed, put into an aluminum packet and frozen in liquid nitrogen. The sampling of root, rhizosphere and soils is described in detail in our previous publications 1, 2. Briefly, roots were removed from the ten plants, mixed together, transferred to 50 ml tubes with detergent-phosphate buffer (6.33 $NaH_2PO_4 \bullet H_2O$ and 8.5 g $Na_2HPO_4 \bullet$anhydrous in 1 L water, autoclaved; cooled, 200µl Silwet-77 added; pre-cooled in ice-water mixture), and vortexed at full speed for 2 min 1. The roots were removed from the tube, the liquid-filled tube was saved, and the roots were transferred to a 200-ml plastic cup with phosphate buffer without detergent (6.33 $NaH_2PO_4 \bullet H_2O$ and 8.5 g $Na_2HPO_4 \bullet$anhydrous in 1 L water, autoclaved; pre-cooled in ice-water mixture), vortexed at full speed for 1 min twice, dried by clean paper towels, put into aluminum packet and frozen in liquid nitrogen 1. The saved, liquid-filled tube containing the rhizosphere was centrifuged at full speed for 3 min, the buffer discarded and the rhizosphere pellet frozen in liquid nitrogen 1. Simultaneously, soil at 6" depth was collected adjacent to the ten sampled plants using 6" soil collection tubes 1. Ten samples were mixed, transferred to a 50-ml centrifuge tube, and frozen in liquid nitrogen 1.
Data collection	DNA was extracted from 1026 samples. ITS2 was amplified and subject to library preparation. Libraries were sequenced by Illumina Miseq PE300 at the Vincent J. Coates Genomics Sequencing Laboratory (GSL, University of California, Berkeley, CA, USA). Raw sequencing were filtered and clustered into OTUs using Usearch. Ling Xu, Cheng Gao, Liliam Montoya extracted DNAs, library preparation and sequencing. Cheng Gao carried out the Bioinformatic analysis.
Timing and spatial scale	Weekly samples of leaf, root, rhizosphere and soil were taken in 2016 for control plots on June 8, 15, 22, 29; July 6, 13, 20, 27; August 3, 10, 17, 24, 31, and September 7, 14, 21, 28. Our research has 18 plots (16m * 8m each) in a 76 m * 56 m field.
Data exclusions	No data was excluded from the analysis
Reproducibility	Our design has three replicates of each treatment
Randomization	Our experiment is a random block design of three replicates. All 1026 DNAs were randomly assigned into four libraries for Illumina sequencing
Blinding	Blinding is not possible for our research

Did the study involve field work? ☒ Yes ☐ No

Field work, collection and transport

Field conditions	Our agricultural filed is located in the Central Valley, a semiarid zone with a mean annual temperature of 17.8°C and mean annual precipitation of 325 mm, almost all of which falls between November and April.
Location	36.6008° N, 119.5109° W
Access and import/export	No permit is required to work on sorghum in California
Disturbance	No disturbance to natural environment

Reporting for specific materials, systems and methods

We require information from authors about some types of materials, experimental systems and methods used in many studies. Here, indicate whether each material, system or method listed is relevant to your study. If you are not sure if a list item applies to your research, read the appropriate section before selecting a response.

Materials & experimental systems

n/a	Involved in the study
☒	☐ Antibodies
☒	☐ Eukaryotic cell lines
☒	☐ Palaeontology
☒	☐ Animals and other organisms
☒	☐ Human research participants
☒	☐ Clinical data

Methods

n/a	Involved in the study
☒	☐ ChIP-seq
☒	☐ Flow cytometry
☒	☐ MRI-based neuroimaging
